# Viral macrodomains: a structural and evolutionary assessment of the pharmacological potential

**DOI:** 10.1098/rsob.200237

**Published:** 2020-11-18

**Authors:** Johannes Gregor Matthias Rack, Valentina Zorzini, Zihan Zhu, Marion Schuller, Dragana Ahel, Ivan Ahel

**Affiliations:** Sir William Dunn School of Pathology, University of Oxford, South Parks Road, Oxford OX1 3RE, UK

**Keywords:** (ADP-ribosyl)hydrolase, alphavirus, antiviral poly(ADP-ribosyl)polymerases, coronavirus, non-structural protein 3, X domain

## Abstract

Viral macrodomains possess the ability to counteract host ADP-ribosylation, a post-translational modification implicated in the creation of an antiviral environment via immune response regulation. This brought them into focus as promising therapeutic targets, albeit the close homology to some of the human macrodomains raised concerns regarding potential cross-reactivity and adverse effects for the host. Here, we evaluate the structure and function of the macrodomain of SARS-CoV-2, the causative agent of COVID-19. We show that it can antagonize ADP-ribosylation by PARP14, a cellular (ADP-ribosyl)transferase necessary for the restriction of coronaviral infections. Furthermore, our structural studies together with ligand modelling revealed the structural basis for poly(ADP-ribose) binding and hydrolysis, an emerging new aspect of viral macrodomain biology. These new insights were used in an extensive evolutionary analysis aimed at evaluating the druggability of viral macrodomains not only from the *Coronaviridae* but also *Togaviridae* and *Iridoviridae* genera (causing diseases such as Chikungunya and infectious spleen and kidney necrosis virus disease, respectively). We found that they contain conserved features, distinct from their human counterparts, which may be exploited during drug design.

## 1. Background

**Figure 1.**
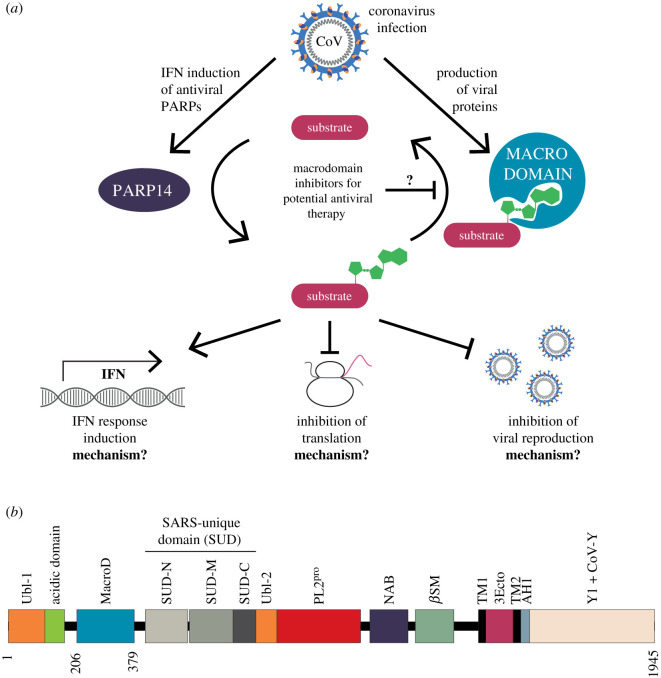
Model for the role of ADP-ribosylation following coronavirus infection. (*a*) Infection of cells with coronavirus leads to the induction of an interferon (IFN) response and the accumulation of human antiviral as well as viral proteins, such as human PARP14 and viral macrodomain, respectively. The ADP-ribosylation activity of PARP14 stimulates maintenance of IFN responsive gene expression, downregulation of translation and prevents viral replication. This is antagonised by the viral macrodomain, part of the multidomain non-structural protein 3 (nsp3), which exhibits (ADP-ribosyl)hydrolase activity and is required for evasion of immune responses and efficient viral replication. However, both the mechanism underlying the changes in cellular processes as well as the precise targets for (de)ADP-ribosylation are currently unknown. (*b*) Schematic overview of the nsp3 domain architecture of SARS-CoV-2. Ubl, ubiquitin-like domain; MacroD, macrodomain of the MacroD-like class; SUD, SARS-unique domain (structurally subdivided into N-terminal, middle and C-terminal SUD with N- and M-SUD harbouring a macrodomain fold and SUD-C a ‘N-terminal domain of CyaY-like' fold); PL2^pro^, papain-like protease 2; NAB, nucleic acid binding domain; *β*SM, betacoronavirus-specific marker; TM, transmembrane region; 3Ecto, Nsp3 ectodomain (also termed ‘zinc-finger domain'); AH1, aliphatic helix 1; Y1 + Y-CoV, C-terminal region of unknown function with the initial domain (Y1) widely conserved and an apparently coronavirus specific (Y-CoV) addition. Domain boundaries were inferred by sequence comparison following a summary by Lei and colleagues [6].

In recent decades, emerging infectious diseases have increased in frequency [1,2], with disease agents of wildlife origin, most importantly RNA viruses, particularly overrepresented [3–5]. Among these, coronaviruses (CoVs) have proven to be significant pathogens of both veterinary and medical importance, and are responsible for the current COVID-19 pandemic as well as two recent epidemics: severe acute respiratory syndrome (SARS) and Middle Eastern respiratory syndrome (MERS). The ability of CoVs to establish infection and to cause disease is dependent on their ability to inhibit the first line of host defence: the innate immune response. Central to this is the interferon (IFN) response which induces an antiviral state in both infected and neighbouring bystander cells by triggering the expression of IFN-stimulated genes. The proteins encoded by these genes fulfil a variety of functions including restricting access to host factors, sensing and degrading of viral RNA, and slowing protein translation (figure 1*a*). ADP-ribosylation, the reversible post-translational modification of proteins and nucleic acids, has recently emerged as a key regulatory mechanism in the establishment of an antiviral environment [7]. The modification reaction consists of the transfer of an ADP-ribose (ADPr) moiety from β-nicotinamide adenine dinucleotide (β-NAD^+^) onto an acceptor site, most commonly an amino acid side chain or a nucleic acid terminus. The process is catalysed by (ADP-ribosyl)transferases (ARTs), including the poly(ADP-ribosyl)polymerases (PARPs, also termed ARTDs) family [8,9]. A subset of PARPs, PARP1, 2 and 5a/b in humans, can extend the initial modification by adding further ADPr units, thus forming linear (ribose(1″ → 2′)ribose) or, less commonly, branched (ribose(1″ → 2″)ribose) polymers (termed poly(ADP-ribose) (PAR)) [8,10]. In humans, the PARP family consists of seventeen members and their function has been associated with the regulation of several fundamental cellular pathways including Wnt signalling, DNA damage repair, protein degradation and stress response [11–14]. The expression of several human PARPs is induced during the IFN response. In particular, PARP7, 9, 10 and 12–15 have been confirmed to play a role in the immune response, and thus are collectively termed the antiviral PARPs [15–20]. These proteins fulfil complex functions, consequently the exact physiological targets and modes of action remain largely elusive.

Currently, the best-characterized role for antiviral PARPs is in the immune response to various RNA viruses, including HIV, Ebola, influenza and coronavirus [21–25]. For instance, PARP13 (also named zinc antiviral protein (ZAP)) binds to viral RNAs and promotes their degradation in a number of RNA viruses, including retroviruses, alphaviruses and filoviruses [15,17,26,27]. Moreover, PARP13 is required for the degradation of the RNA polymerase subunits PA and PB2 of the type A influenza virus [28]. Similarly, PARP9 targets the 3C protease of encephalomyocarditis virus (EMCV) for degradation [19]. PARP12 inhibits the replication of Zika virus by ADP-ribosylation of the viral NS1 and 3 proteins [18] and has also been implicated in preventing replication of vesicular stomatitis virus (VSV), Venezuelan equine encephalitis virus (VEEV), Rift Valley fever virus (RVFV) and EMCV [15,29]. In response to CoVs, PARP14 promotes antiviral pro-inflammatory cytokine production by increasing interleukin 4 (IL-4)-dependent transcription activation, while PARP9 is shown to enhance IFN signalling by altering the host histone modification pattern, which ultimately suppresses viral replication [19,30]. A recent study provided further evidence that the main PARPs for the defence against CoVs are PARP12 and PARP14 [31].

Unsurprisingly, viruses have evolved compensatory mechanisms to counteract antiviral PARP function, preventing creation of an antiviral environment [7,21,32–34]. The genomes of members of the *Coronaviridae*, *Togaviridae* (e.g. VEEV and Chikungunya virus (CHIKV)) and *Iridoviridae* (e.g. infectious spleen and kidney necrosis virus (ISKNV) and red sea bream iridovirus (RSIV)), as well as rubella virus and hepatitis E virus, encode a macrodomain either as a self-standing protein or as part of a multidomain protein. Evolutionarily, these viral macrodomains fall within the MacroD-type class, which is primarily composed of (ADP-ribosyl)hydrolases that are able to cleave the protein-ADPr bond [35]. Indeed, catalytic activity of several viral macrodomains from CoVs, alphaviruses and HEV has been experimentally confirmed using model ADP-ribosylated substrates [21,32–34,36–40].

In *Coronaviridae*, including the betacoronavirus SARS-CoV-2, the causative agent of the COVID-19 pandemic, the macrodomain is part of non-structural protein 3 (nsp3), a 200 kDa multidomain protein encoded as part of open reading frame 1 (ORF1; figure 1*b*). The hydrolase activity of the nsp3 macrodomain was shown to be crucial for pathogenesis of several coronavirus strains *in vivo*, including murine hepatitis virus (MHV), human coronavirus 229E (HCoV-229E) and SARS-CoV [41]. Furthermore, the macrodomain was linked to promoting viral replication and a strong immune evasive function. In bone-marrow-derived macrophage and murine models of coronavirus infection, catalytic mutation or deletion of the macrodomain leads to poor viral replication; lower viral load in target organs, including lungs (SARS-CoV), brain and liver (MHV) and general mild disease progression [21,42,43]. Conversely, macrodomain activity appears to be dispensable for viral replication in cell culture for the 17Cl-1, DF-1, MRC-5, L929 and Vero E6 cell lines tested so far [21,42–46]. Recent work supports these findings, showing that impairment of viral replication is driven by PARP12 and 14 and that knockdown of these genes led to partial restoration of pathogenicity of macrodomain-mutant MHV [31]. Grunewald and colleagues suggest that induction of the IFN response is crucial to suppress viral replication, since both PARP12 and 14 are IFN-stimulated genes. Additionally, knockout of the IFN receptor rescued the sensitization of mutant viruses to IFN treatment both *in vivo* and in cell culture [31]. Taken together these findings suggest that the coronavirus macrodomain is a suitable drug target with great potential for antiviral therapy (figure 1*a*).

MacroD-like macrodomains are widely distributed in all kingdoms of live not just in viruses, with two homologues present in humans (*h*MacroD1 and 2). Therefore, evolutionary conservation may limit their suitability as antiviral therapy targets. To tap into the therapeutic potential of coronavirus macrodomain inhibition cross-reactivity of candidate compounds with host macrodomains needs to be suppressed. In this study, we analysed the active site of SARS-CoV-2 macrodomain (S2-MacroD) and compared the ligand-binding properties of the coronavirus macrodomain active site with related viruses and MacroD-like enzymes in general. We used a structural phylogenetic approach to highlight commonalities and differences in the ligand-binding cleft that can be exploited during drug development. To this end, we obtained the crystal structure of the S2-MacroD bound to ADP-ribose and its analogues ADP-HPD and ADP-HPM (characterized as potent inhibitors of the related poly(ADP-ribosyl)glycohydrolase (PARG) enzyme). We identified and characterized the structural basis for the observed functional diversification of MacroD-like enzymes and provide for the first time direct evidence for the hydrolysis of PARP14-ADP-ribosylated substrates. These data suggest that SARS-CoV-2 contains a conserved, enzymatically active macrodomain that can potentially counter the host antiviral ADP-ribosylation process catalysed by PARP14, as well as alter poly(ADP-ribose) (PAR) signalling, thereby enabling viruses to escape the innate immune response of the host. Finally, through identification of the amino acids responsible for the viral macrodomain hydrolase activity, we highlight sites that can be targeted by small-molecule compounds aiming to inhibit viral macrodomain activity as a viable therapeutic approach.

## 2. Methods

### 2.1. Autoradiography and immunoblot analysis and antibodies

Reactions for analysis were stopped by adding four times LDS sample buffer and incubation for 5 min at 90°C. Subsequently, the samples were resolved by SDS-PAGE and either Coomassie Brilliant Blue (CBB) stained and vacuum dried for autoradiography or transferred onto a nitrocellulose membrane. Immunoblot analyses were carried out using primary and secondary antibodies as indicated. Proteins were detected by enhanced chemiluminescence (Pierce). Antibodies used in this study are: horseradish peroxidase-conjugated chicken avidin (abcam), mouse monoclonal ethenoadenosine antibody [1G4] (Novus Biologicals; RRID: AB_350711), anti-pan-ADP-ribose binding reagent MABE1016 (Merck-Millipore; RRID: AB_2665466), secondary swine anti-rabbit (Dako; RRID: AB_2617141) and secondary goat anti-mouse antibodies (Dako; RRID: AB_2617137).

### 2.2. Plasmid construction

Expression vectors for PARP14-WWE-CAT, PARP14-MOD1, MOD2 and MOD3 were described previously [47,48]. The sequences of the SARS-CoV-2 macrodomain (S2-MacroD; GenBank: YP_009725299; aa residues 206–379 of nsp3) together with an N-terminal HRV3C cleavage site were codon-optimized for expression in *E. coli*, gene synthesized (GeneArt; Thermo Fisher Scientific) and cloned into pDONR221 using the Gateway technology (Thermo Fisher Scientific). Subsequently, the gene was transferred into pDEST17 for expression. All indicated mutations were introduced via PCR-based site-directed mutagenesis.

### 2.3. Protein expression and purification

#### 2.3.1. For biochemistry

Expression of recombinant S2-MacroD in Rosetta (DE3) cells was induced at OD_600_ of 0.6 with 0.4 mM isopropyl β-D-1-thiogalactopyranoside (IPTG). Cells were grown overnight at 18°C in LB medium supplemented with 2% (w/v) D-glucose, 2 mM MgSO_4_ and appropriate antibiotics and harvested by centrifugation. Recombinant His-tagged S2-MacroDs (wild-type and mutants) were purified at 4°C by Ni^2+^-NTA chromatography (Jena Bioscience) according to the manufacturer's protocol using the following buffers: all buffers contained 50 mM Tris–HCl (pH 8) and 500 mM NaCl; additionally, lysis, wash and elution buffers contained 25 mM, 40 mM and 500 mM imidazole, respectively. All proteins were dialysed overnight against 50 mM Tris–HCl (pH 8), 200 mM NaCl, 1 mM dithiothreitol (DTT) and 5% (v/v) glycerol. Purity of the protein preparations was assessed using SDS-PAGE and CBB staining and aliquots were stored at −80°C until use.

PARP14 macrodomain proteins (PARP14 MOD1, MOD2 and MOD3) were produced by transforming corresponding plasmids into BL21(DE3)-R3-pRARE cells and grown at 37°C in LB medium supplemented with appropriate antibiotics until OD_600_ 0.5–0.6, then cooled to 18°C and supplemented with 0.5 mM IPTG at an OD_600_ of 0.8 to induce protein expression overnight. After harvesting by centrifugation, cell pellets were resuspended in lysis buffer (50 mM HEPES [pH 7.5], 500 mM NaCl, 20 mM imidazole, 5% glycerol, 0.5 mM tris(2-carboxyethyl)phosphine (TCEP), 1:2000 Calbiochem protease inhibitor cocktail set III) and lysed by sonication. Proteins were purified by Ni^2+^-NTA chromatography (Jena Bioscience) and eluted stepwise in binding buffer with 40–250 mM imidazole. PARP14 MOD1 was additionally purified by ion-exchange chromatography using a HiTrap 5 ml SP HP (GE Healthcare) equilibrated in 25 mM HEPES (pH 7.5), 75 mM NaCl, 0.5 mM TCEP. All proteins were dialysed overnight against 50 mM HEPES (pH 7.5), 300 mM NaCl, 5% glycerol, 0.5 mM TCEP. Purity of the protein preparations was assessed using SDS-PAGE and CBB staining and aliquots were stored at −80°C until use.

PARP14 WWE-CAT domain was produced in Rosetta (DE3) cells transformed with the corresponding plasmid by growing them to an OD_600_ of 0.6 in 2xYT medium supplemented with appropriate antibiotics. After expression induction with 0.5 mM ITPG, cells continued growing at 18°C overnight before harvesting. Pellets were resuspended in lysis buffer (100 mM HEPES [pH 8], 500 mM NaCl, 10% (v/v) glycerol and 0.5 mM TCEP) supplemented with protease inhibitor (Roche) and benzonase. The cells were lysed by high-pressure homogenization and the protein purified from the lysate by Ni^2+^-NTA chromatography (Jena Bioscience). Protein was eluted with incremental imidazole concentration (10–400 mM) in lysis buffer. Fraction containing PARP14 WWE-CAT were pooled and salt concentration adjusted to 200 mM NaCl by addition of 100 mM HEPES (pH 7.5), 10% (v/v) glycerol and 0.5 mM TCEP prior to binding on a Heparine column (GE Healthcare). The flow-through containing PARP14 WWE-CAT was collected, concentrated and loaded on a HiLoad Superdex 75 pg column. The protein was eluted using 20 mM HEPES (pH 7.5), 300 mM NaCl and 0.5 mM TCEP. Protein fractions were pooled, concentrated and 10% (v/v) glycerol was added before storage at −80°C.

#### 2.3.2. For crystallization

S2-MacroD was expressed as described above and pellets were resuspended in 50 mM HEPES (pH 7.8), 500 mM NaCl, 20 mM imidazole, 0.2 mM TCEP and 10% (v/v) glycerol. Cell were treated for 1 h with benzonase and egg white lysozyme, before high-pressure homogenization. The soluble protein was affinity purified over a HisTrap HP column (GE Healthcare), followed by dialysis against lysis buffer in the presence of HRV3C protease for proteolytic cleavage of the His-tag. Rebinding to a HisTrap HP column and a GSTrap 4B Column (GE Healthcare) was used to remove uncleaved protein and HRV3C protease, respectively. The final step involved size exclusion chromatography using a HiLoad Superdex 75 pg column with 10 mM MOPS (pH 7.2), 100 mM NaCl and 1 mM DTT as elution buffer.

### 2.4. Enzymatic assays

#### 2.4.1. PARP14 catalysed ADP-ribosylation

PARP14 automodification was carried out using 1 µM recombinant PARP14 (WWE-CAT) in assay buffer (50 mM Tris–HCl (pH 7.5), 200 mM NaCl, 1 mM MgCl_2_ and 1 mM DTT) supplemented with 40 µM β-NAD^+^ and 0.3 µCi ^32^P-NAD^+^. Reactions were carried out for 60 min at 30°C. For trans-modification of the PARP14 macrodomains, 1 µM of indicated proteins were added to the above reaction. Reactions using NAD^+^-analogues were carried out as described above using 50 µM of etheno-NAD^+^ (Sigma), biotin-NAD^+^ (Tocris) or β-NAD^+^ (Sigma), respectively.

#### 2.4.2. (ADP-ribosyl)hydrolase assays

To test the (ADP-ribosyl)hydrolase activity of S2-MacroD, PARP14 was modified with NAD^+^ or its analogues as described above. The initial reactions were supplemented with 1 µM S2-MacroD, either wild-type or mutant protein as indicated, and the reactions were allowed to continue for 45 min at 30°C. Reactions were stopped with LDS sample buffer (Life Technologies) and incubation at 95°C for 3 min. Samples were then analysed by SDS-PAGE, immunoblot and autoradiography as appropriate.

### 2.5. Crystallization

S2-MacroD for crystallization was expressed and purified as described above. The protein was then concentrated to 550 µM (10.4 mg ml^−1^). Initial S2-MacroD crystals grew in the presence of a 7.5-molar excess of ADPr in 2.1M DL-malic acid (pH 7) using a 1:1 mother liquor (ML) to protein ratio. The crystallization condition was optimized by adjusting the concentration of protein and DL-malic acid, varying the protein-to-ML ratio, as well as using the JBScreen Plus HTS additive screen (Jena Bioscience). The final growth conditions for the datasets reported here are as follows. (i) ADPr bound form (PDB 6Z5T): 886 µM S2-MacroD containing 6.645 mM ADPr mixed with ML (1.775M DL-malic acid (pH 7), 10 mM TCEP) in a 1:3 ratio. Crystals were cryoprotected using 50% saturated sodium malonate (pH 7.2). (ii) ADP-HPD bound form (PDB 6Z6I): 550 µM S2-MacroD containing 2.75 µM ADP-HPD (Merck Millipore) mixed with ML (1.9M DL-malic acid, 5% (v/v) glycerol) in a 1:1 ratio. Crystals were cryoprotected using 18% (v/v) ethylene glycol in ML. (iii) ADP-HPM bound form (PDB 6Z72): 550 µM S2-MacroD, 4.125 mM ADP-HPM (synthesis described in [49]) mix with ML (1.775M DL-malic acid (pH 7), 4.5% (v/v) ethylene glycol, 200 mM potassium cyanate) in a 1:1 ratio. Crystals were cryoprotected using 18% (v/v) ethylene glycol in ML. All crystals were cryoprotected by submerging them into indicated cryoprotectant solutions for 5 s prior to vitrification in liquid nitrogen.

### 2.6. X-ray data collection, processing and refinement

The X-ray data were collected to a resolution of 1.57 Å (ADPr; PDB 6Z5T), 2.0 Å (ADP-HPD; PDB 6Z6I) and 2.3 Å (ADP-HPM; PDB 6Z72) at beamline I03 of the Diamond Light Source (Rutherford Appleton Laboratory, Harwell, UK), using an Eiger2 XE 16M detector. Upon collection, these data were indexed, integrated and scaled automatically using Xia2 [50] and merged with Aimless [51]. Data collection statistics are given in electronic supplementary material, table S1. The phase problem was solved by molecular replacement with PHASER [52] as implemented in the CCP4i2 package [53]. The *apo* SARS-CoV macrodomain structure (PDB 2ACF; [36]) was used as search model for the molecular replacement of S2-MacroD:ADPr and the refined model was subsequently used as a search model for the molecular replacement of the other structures reported here. The molecular replacement solutions were refined by iterative cycles of manual structure building using Phenix refinement [54] and Coot [55]. For all structures automatically generated local non-crystallographic symmetry (NCS) restraints were applied. At the end of the refinement, water molecules and other ligands (i.e. ethylene glycol, glycerol etc.) were included in the models where relevant. Translation/Libration/Screw (TLS) parameters (one TLS group per protein chain) were also included in the refinement where appropriate. Structure validation was conducted using MolProbity [56]. The final refinement statistics are given in electronic supplementary material, table S1.

### 2.7. Modelling of PAR dimer onto S2-MacroD

We used the 6Z6I structure of the S2-MacroD domain in complex with ADP-HPD as a template for building a model of S2-MacroD:PAR dimer complex. The two molecules of ADP-HPD visible in the crystal structure guided the placement of 90% of the PAR dimer. Two molecules of ADP ribose were designed in ChemDraw v. 19 (PerkinElermers Informatics) and bonded together via the 2′OH of the first ADP ribose molecule with C1″ of the second ADP ribose molecule. The SMILE string of the generated PAR dimer was converted to 3D ligand via Ligand Builder in Coot [55] and fitted into the density following the orientation of the two ADP-HPD molecules crystallized. The structure was further energy minimized in Chimera [57]. All atoms were included in the energy calculation. The parameters used are as follows: 10 steps of steepest descent minimization (0.02 Å step size), 10 steps of conjugate gradient (0.02 Å step size), 10 of update interval.

### 2.8. Modelling of PAR dimer onto S2-MacroD homologues structures

The PAR dimer from S2-MacroD:PAR dimer complex was transferred to 3GQO, 3EWR and 4IQT by superposition with PDB entry 6Z6I. The resulting complexes were then energy minimized in Chimera [57] to resolve clashes. All atoms were included in the energy calculation. The parameters used are as follow: 10 steps of steepest descent minimization (0.02 Å step size), 10 steps of conjugate gradient (0.02 Å step size) and 10 of update interval.

### 2.9. *In silico* analysis of the viral macrodomains

#### 2.9.1. Inference of phylogenetic relationships and sequence similarities

Macrodomains sequences from all kingdoms of life and viruses (electronic supplementary material, table S2) were extracted from their sequential context based on Clustal Omega alignment [58] using crystallographic data to determine domain boundaries. Final alignments were generated using JalView v. 2.11 [59] and the Mafft L-INS-i algorithm integrated therein [60]. The evolutionary histories of betacoronavirus and all MacroD-like sequences were inferred by using the maximum-likelihood method and Le_Gascuel_2008 model [61] with an automatically obtained initial tree for the heuristic search by applying the maximum-parsimony method. The analysis of all MacroD-like sequences was carried out using a site coverage of 95% with partial-deletion option. Confidence levels were estimated using 1000 cycles of the bootstrap method. Evolutionary analyses were conducted in MEGA X [62].

To analyse the relationship between *Iridoviridae* and host-derived macrodomains, we extracted coding sequences of *Iridoviridae*, their fish hosts, other *Animalia, Fungi* and *Plantae* from the sequential context (electronic supplementary material, table S3). Macrodomain boundaries were derived in relation to the above-used protein sequences for ISKNV and *h*MacroD1 for *Iridoviridae* and fish-host sequences, respectively. Final alignments were generated using JalView v. 2.11 [59] and the Mafft L-INS-i algorithm integrated therein [60]. The evolutionary history was inferred by using the maximum-likelihood method and a general time-reversible model [63] with a discrete γ distribution and allowing for evolutionary invariant sites (+G+I). Initial tree for the heuristic search was obtained automatically by applying the maximum-parsimony method. The analysis accounted for codon positions and was carried out using a site coverage of 95% with partial-deletion option. Confidence levels were estimated using 1000 cycles of the bootstrap method. Evolutionary analyses were conducted in MEGA X [62].

Pairwise identity and similarity were determined using the Needleman–Wunsch algorithm implemented as part of the EMBL-EBI search and sequence analysis server [64].

Alignment representations were created with JalView v. 2.11 [59].

#### 2.9.2. Analysis of natural variants of S2-MacroD

For the analysis of natural variant within the S2-MacroD we used the natural selection analysis (http://covid19.datamonkey.org) of the covid.galaxyproject [65]. The sequence data for this project were provided by GISAID and analysed using HyPhy to inferred codons evolving non-neutrally using MEME and/or FEL methods, restricted only to internal tree branches, or carried a high minor allele frequency (MAF > 0.2) among unique haplotypes. Detected residues are classified as ‘pervasive positive' if the site has on average a dN/dS > 1 along interior tree branches and is accumulating non-synonymous changes, ‘episodic' if only a fraction of interior branches has dN/dS > 1, and ‘pervasive negative' if the site has on average a dN/dS < 1 along interior tree branches. This method may overlook perfectly conserved sites. Detected variants within S2-MacroD were investigated with respect to their position within the structure and potential structural consequences associated with the change (table 1).

**Table 1.** Natural selection analysis of S2-MacroD and prediction of functional consequences.

residue	variant(s)^a,b^	total no. variants	no. of sequences^c^	occurrence^c^	conservation score^d^	classification^e^	species^b^
**pervasive positive**							
Asp218	Glu(325), Asn(8), Tyr(2), Gly(1)	336	75 640	yes	2	N, SE	Glu: Bat-CoV-HKU9, HCoV-EMC, *h*MacroD1, *h*MacroD2 Asn: *Afu*MacroD, Bat-CoV-HKU4 Tyr: *n.f*. Gly: *Cel*MacroD, EriCoV-1, FCoV, RuV, TGV
Pro340	Ser(108), Leu(81)	189	75 664	yes	1	N	Ser: ChRCoV-HKU24, HCoV-OC43, *Mgr*MacroD Leu: *Afu*MacroD, *Afu*YmdB, FCoV, HCoV-229E, TGV
Gly282	Val(97), Cys(12), Ser(4)	113	75 646	no	3	N	—
His295	Tyr(53), Arg(4)	57	75 666	yes	1	N, SE	Tyr: *Afu*YmdB, *Dpp*MacroD, *h*MacroD1, *h*MacroD2, ISKNV, *Lch*MacroD, *Lis*YmdB, SACIV, TRBIV Arg: *Ath*MacroD, *Atr*MacroD, HCoV-229E, *Ppa*MacroD
Thr350	Ile	55	75 659	yes	3	N, SE	SARSr-CoV-HKU3-13
Thr237	Ile(19), Ala(6), Pro(4)	29	75 667	yes	3	N, SE	Ile: *n.f*. Ala: Bat-CoV-HKU9 Pro: Hp-betaCoV
Ala333	Val	17	75 665	yes	6	N	Bat-CoV-512, FCoV, TGV
Leu292	Phe	13	75 667	no	0	D^f^ (Phe)	—
Asp309	Asn(3), Gly(2)	5	75 668	yes	0	N	Asn: HEV Gly: EEEV, GETV, ONNV, RuV, RRV, SDV, TONV, VEEV, WEEV
Ala243	Val	4	75 669	no	11	A^g^	—
**episodic**							
Glu206	Lys(23), Ile(1)	24	75 666	yes	gap	N, SE	Lys: *n.f*. Ile: *Dpp*MacroD, RoBat-CoV GCCDC1
Asn276	Arg(6), Ile(1), Thr(1), Ser(1)	9	75 664	yes	gap	N, SE	Arg: *Ath*MacroD, Ch*R*CoV-HKU24 Ile: *n.f*. Thr: Bulbul-CoV Ser: FCoV, PDCoV, *Ppa*MacroD
**pervasive negative**							
Leu287^h^	*n.a.*	*n.a.*	75 669				
Leu326^h^	*n.a.*	*n.a.*	75 669				
Leu357^h^	*n.a.*	*n.a.*	75 669				
Val228^h^	*n.a.*	*n.a.*	75 669				
Ala254^h^	*n.a.*	*n.a.*	75 669				
Ser332^h^	*n.a.*	*n.a.*	75 668				
Glu229	Gly(7), Asp(2), Ala(1)	10	75 668	yes	2	N, SE	Gly: GETV, RRV Asp: ChRCoV-HKU24, RuV Ala: Bat-CoV-512
Ala242	Val	5	75 665	no	7	A^i^	—
Leu297	Phe(3), Ile(1)	4	75 663	yes	7	D^f^ (Phe)	Phe: *Afu*YmdB, HCoV-229E, HCoV-NL63 Ile: *Afu*MacroD, *Ath*MacroD, *Atr*MacroD, *Cel*MacroD, *Dpp*MacroD, EEEV, GETV, HEV, *h*MacroD1, *h*MacroD2, ISKNV, *Lis*YmdB, *Mma*YmdB, *Mgr*MacroD, ONNV, *Ppa*MacroD, RRV, RuV, SACIV, SDV, TONV, TRBIV, VEEV, WEEV
Asn305	Asp(1), Tyr(1), Ile(1), Ser(1)	4	75 661	yes	2	N, SE	Asp: *Ath*MacroD, *Atr*MacroD Tyr: *Cel*MacroD, *h*MacroD1 Ile: *n.f*. Ser: *Afu*YmdB, EEEV, GETV, ONNV, PDCoV, RRV, SACIV, TRBIV, WEEV
Pro302	Gln	3	75 668	no	10	D^f^	—
Val355	Ala	3	75 668	no	7	N	—
Asp366	Asn(2), Glu(1)	3	75 666	yes	1	N, SE	Asn: Bat-CoV-HKU4, *Ppa*MacroD, Glu: *Afu*YmdB, Bat-CoV-HKU9, BtRI-betaCoV, *Cel*MacroD, EriCoV-1, *Lch*MacroD, *Mgr*MacroD, SACIV, SARS-CoV, TRBIV, Whale-CoV-SW1
Leu257	Ile(1), Phe(1)	2	75 669	yes	9	D^f^ (Phe)	Ile: *Afu*MacroD, *Afu*YmdB, *Ath*MacroD, *Atr*MacroD, Bat-CoV-HKU4, Bulbul-CoV, *Cel*MacroD, Ch*R*CoV-HKU24, *Dpp*MacroD, *Eco*YmdB, EriCoV-1, FCoV, HCoV-EMC, HCoV-HKU1, HCoV-NL63, HCoV-OC43, *h*MacroD1, *h*MacroD2, Hp-betaCoV, IBV, ISKNV, *Lch*MacroD, *Lis*YmdB, MHV-A59, *Mgr*MacroD, *Mma*YmdB, *Ppa*MacroD, PDCoV, RuV, SACIV, TGV, TRBIV Phe: HEV

^a^No. of occurrences given in parenthesis.

^b^*n.a.,* not applicable*; n.f.,* not found*.*

^c^No. of sequences used for codon analysis (variability due to sequencing quality).

^d^occurrence and conservation score based on full alignment (electronic supplementary material, figure S2); conservation scores 11 and 10 are reported on the full alignment as * and +, respectively; gap: insertion/deletion in the sequence alignment.

^e^N, neutral; D, destabilizing (main destabilizing variant given in parenthesis); A, ADPr binding interface; SE, surface-exposed (defined using the findSurfaceResidues module in PyMOL with a cut-off value of 2.5 Å^2^, i.e. atoms with > cut-off Å^2^ exposed to solvent are considered exposed).

^f^The protein variants L257I/F, L292F, L297I/F and P302Q will most likely destabilize the structure/folding of the protein due to clashing with neighbour residues, with only slight impact expected for the Leu-to-Ile variants due to the comparable physico-chemical properties.

^g^Ala243 is part of the conserved NAAN motif and situated in the catalytic pocket. It is the α-face of the distal ribose. The increase in Van der Waals volume associated with the A243V variant may destabilize the ligand binding.

^h^Sequences were picked up due to the occurrence of silent mutations.

^i^Ala242 is part of the conserved NAAN motif situated in the catalytic pocket. It is facing the ADPr phosphates. The distance between the A242V variant and the phosphates is reduced from 3.5–4.3 Å to 2.1–2.9 Å, most likely destabilizing the ligand binding.

#### 2.9.3. Analysis of macrodomains found in the human-associated microbiome

The human biome deposited in the MGnify database [66] was searched using the S2-MacroD amino acid sequence. A total of 1747 sequences were identified and aligned using Clustal Omega [58]. The sequences were manually inspected for quality and gaps in the macrodomain catalytic region, resulting in 41 sequences being discarded from the analysis. The remaining sequences were re-aligned using Clustal Omega and analysed for the presence of the three catalytic motives identified among the different MacroD subclasses (figure 7 and text for details).

## 3. Results and discussion

### 3.1. S2-MacroD can reverse PARP14-derived ADP-ribosylation

**Figure 2.**
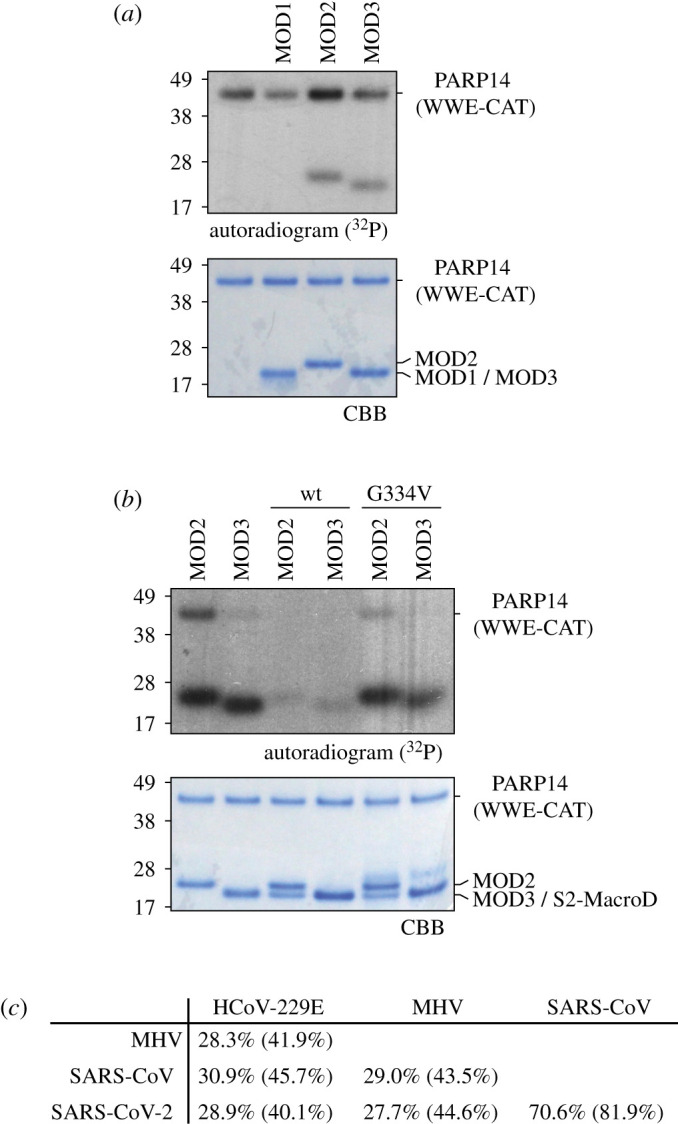
S2-MacroD reverses PARP14-derived ADP-ribosylation. (*a*) PARP14 can efficiently modify itself *in vitro*. *Auto*- and *trans*-ADP-ribosylation activity (PARP14 WWE-CAT and PARP14 macrodomains 1–3 [MOD1-3], respectively) of PARP14 were assessed by incubation with ^32^P-NAD^+^*in vitro.* Samples were analysed by SDS-PAGE followed by CBB staining and autoradiography (^32^P) which reveal efficient automodification of PARP14 WWE-CAT as well as trans-modification of MOD2 and MOD3. (*b*) S2-MacroD can reverse PARP14-derived ADP-ribosylation. PARP14 WWE-CAT, MOD2 and MOD3 were modified as in (*a*) followed by incubation with S2-MacroD. Samples were analysed by CBB staining and autoradiography and show that S2-MacroD wild-type (wt) can efficiently remove ADP-ribosylation from all three proteins. (*c*) Pairwise sequence identity comparison of coronaviral MacroD domains with experimentally proven (ADP-ribosyl) hydrolase activity. Sequence identity and similarity (in parentheses) are provided.

PARP14 has emerged as an important cellular factor in the restriction of coronaviral infections and the available data strongly suggest that this function requires (ADP-ribosyl)transferase activity [31]. Experimental evidence shows that PARP14 is able to automodify *in vitro* [67]. Furthermore, recent high-throughput mass-spectrometry revealed modification sites within the macrodomains (MOD1–3) and the WWE domain of PARP14 isolated from cells [68], but whether the latter modifications originate from automodification or from modification in *trans* by some other (ADP-ribosyl)transferases present in human cells remained elusive. We, therefore, tested the activity of the catalytic fragment of PARP14, consisting of WWE and catalytic domain (WWE-CAT), on isolated MODs. As expected, the WWE-CAT fragment automodifies, but also demonstrated efficient modification of MOD2 and MOD3 (figure 2*a*). Next, we evaluated whether S2-MacroD has the ability to remove the modification from these new substrates. Our *in vitro* enzymatic assay, indeed, shows that S2-MacroD removed ADP-ribosylation from PARP14 WWE-CAT, MOD2 and MOD3 (figure 2*b*). This shows that S2-MacroD retains catalytic activity despite its evolutionary divergence from other betacoronaviruses and coronavirus MacroDs with confirmed enzymatic activity (figure 2*c*; electronic supplementary material, table S4) [21,32,33]. Together with the finding that PARP14 and other antiviral PARPs are under positive natural selection [20,69], this is indicative of the participation of coronaviral macrodomains in the host–virus arms race and suggests that their sequences are selected for diversity while maintaining the enzymatic function.

### 3.2. Structure determination of S2-MacroD

**Figure 3.**
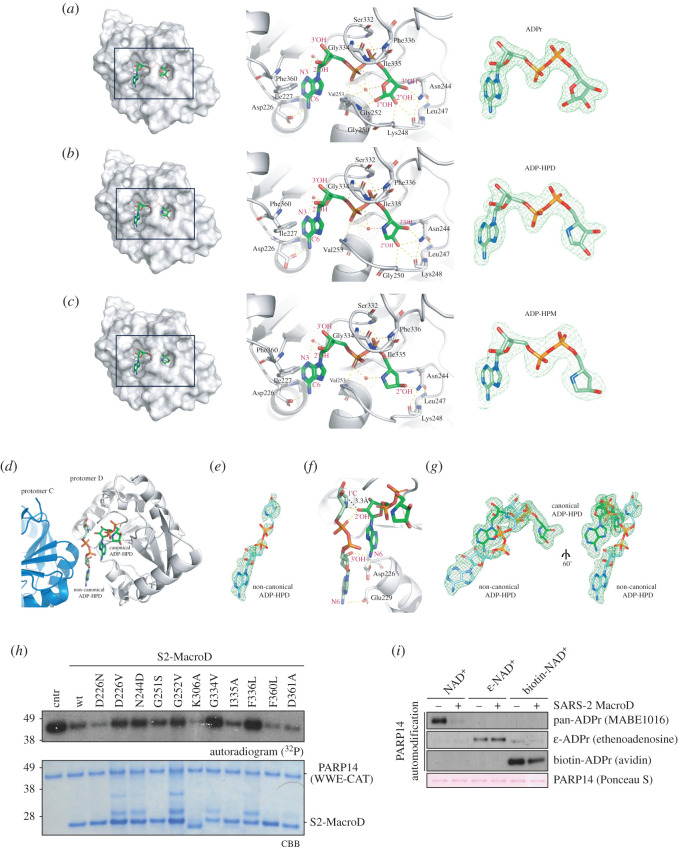
Identification of key residues for ligand binding, catalysis and drug design. (*a*) Left panel: Surface representation of S2-Macro:ADPr complex showing the tight coordination of ADPr (green) within a deep binding cleft. Middle panel: Ribbon-liquorice representation of ADPr coordination. Residues (black labels) and ADPr atoms (red labels) involved in the interactions are highlighted. Structural waters are given as red spheres and polar interactions as dotted lines. Right panel: Composite omit map (green) of the ADPr ligand contoured at 2*σ*. (*b*) As (*a*) for the S2-MacroD:ADP-HPD complex. (*c*) As (*a*) for the S2-MacroD:ADP-HPM complex. (*d*) Ribbon representation of the S2-MacroD:ADP-HPD complex. An extra ADP-HPD ligand (non-canonical, light green) is visible between chain C (blue) and chain D (white). The canonical ADP-HPD is shown in dark green sticks. (*e*) Composite omit map of the second bound ADP-HPD ligand contoured at 2*σ*. (*f*) Ribbon-liquorice representation of the interaction between the two ADP-HPD ligands within protomer D. Polar protein-ligand and ligand–ligand interactions are shown in dashes lines (yellow). Measured distance (3.3 Å) between the C1″ atom of the non-canonical ligand and the 2′OH moiety of the canonical ligand is shown as black line. Ligand atoms are labelled in red and S2-MacroD residues in black. (*g*) Composite omit map of both ligands contoured at 2*σ* and shown in two different orientations. (*h*) S2-MacroD variants carrying amino acid substitutions were analysed for their effect on (ADP-ribosyl)hydrolase activity. The model substrate PARP14 WWE-CAT was modified to completion in presence of ^32^P-NAD^+^ and subsequently incubated with either wt or S2-MacroD mutants as indicated. Samples were analysed by CBB staining and autoradiography. (*i*) Substitutions at the adenosine base affect S2-MacroD (ADP-ribosyl)hydrolase activity. The model substrate PARP14 WWE-CAT was modified in the presence of either β-NAD^+^, biotin-NAD^+^ or ε-NAD^+^ as indicated. The reactions were subsequently incubated in the absence or presence of S2-MacroD as indicated. Samples were blotted onto a nitrocellulose membrane and analysed by Ponceau S staining and substitution specific detection reagents.

In order to identify how sequence alterations affect the structure and substrate recognition of S2-MacroD, we solved its protein structure in the presence of the reaction product ADP-ribose (ADPr) by X-ray crystallography (electronic supplementary material, table S1). Additionally, structures were obtained containing the inhibitory analogues ADP-HPD and ADP-HPM (electronic supplementary material, table S1). The protein crystallized in two different space groups depending on the co-crystallized ligand. S2-MacroD2:ADPr crystallized in *P*2_1_2_1_2_1_ with two ligand-bound molecules in the asymmetric unit and was refined to a resolution of 1.57 Å (figure 3*a*). The resulting structure of S2-MacroD:ADPr follows the previously observed fold of a tightly packed *α*/*β*/*α* sandwich with a central six-stranded, mixed β-sheet flanked by six α-helices [35,70]. The ADPr ligand lies within a deep cleft on the crest of the domain. The sequence of the crystallized protein covers the full sequence of the S2-MacroD (representing aa 206–379 of nsp3) with the exception of the two extreme N-terminal residues derived from the purification tag, which are not resolved in the electron density. The two protomers deviate only slightly from each other (r.m.s.d. of 0.091 Å over 149 C*^α^*). On the other hand, S2-MacroD:ADP-HPD and S2-MacroD:ADP-HPM crystallized in *P*2_1_ with four ligand-bound molecules in the asymmetric unit and were refined to a resolution of 2.0 and 2.3 Å, respectively (figure 3*b*,*c*). The overall fold is identical to the S2-MacroD:ADPr structure with r.m.s.d. values of 0.155 Å over 155 C*^α^* and 0.152 Å over 149 C*^α^*, respectively.

### 3.3. Binding of ADPr and its analogues

ADPr is bound within a deep cleft and its overall positioning resembles previously solved structures of MacroD-like enzymes [32,34,35,70–72]. The adenosine moiety lies parallel to the protein surface and is partially shielded by Phe360, which interacts edge-to-face with the aromatic ring system (figure 3*a*). This contrasts with earlier reported structures containing similarly placed aromatic residues (e.g. Phe224 in *h*MacroD2; PDB 4IQY) forming π–π interactions with the adenosine ring. The amino group at the C6 position is interacting with Asp226. The ring is further held in place by interactions between N1 and the backbone of Ile227 as well as through a highly coordinated water molecule (w548) bridging among others N3 with the in-ring oxygen of the proximal ribose. The 2′ and 3′ hydroxyl groups of the latter are rotated out of the binding pocket and make only water-mediated contacts to the protein. The phosphodiester is interacting with several backbone amide groups within a loop region spanning the edge of the binding cleft (Ser332 to Phe336), which was therefore termed the pyrophosphate binding loop [32,73]. In addition, aliphatic side chains of residues within the loop close on top of the binding cleft, thus further restricting the phosphodiester. Up to this point, the coordination of ADP-HPD and –HPM is identical to ADPr (figure 3*b*,*c*). However, in ADPr and ADP-HPD, the distal ribose is bound in a slightly strained position, forced through steric hindrance imposed by Phe336 and stabilized via a structural water molecule (w537; figure 3*a*,*b*). The latter bridges P*^α^* with the in ring oxygen as well as the β-anomeric 1″OH. Two characteristic motifs contribute further to the coordination of the distal ribose, first, the NAAN motif (Asn241 to Asn244 in S2-MacroD) lines the floor of the binding cleft and Asn244 coordinates both the 2″ and 3″OH moieties of the distal ribose (electronic supplementary material, figure S1). In addition, Asn244 interacts also with the backbone amides of Leu247 and Lys248 restraining the flexibility of the catalytic loop, which may influence the positioning of both the distal ribose as well as the linked protein part of substrate. Second, a triple glycine stretch makes further contacts to the 1″ and 2″OH positions via the backbone amide group of Gly252 and Gly250, respectively. These residues are part of a loop region found to be important for catalytic activity, and hence was termed the catalytic loop [32,73]. In contrast to this tight coordination, the other two S2-MacroD structures in complex with ADP-HPD and ADP-HPM show a different pattern of interactions, mainly due to the absence of OH substituents on the pyrrolidine ring (figure 3*b*,*c*). ADP-HPD, which retains the 3′OH interaction typical of the ADPr, is, excepting the missing 1″ OH interactions, indistinguishably coordinated compared to ADPr (figure 3*b*), whereas ADP-HPM, which lacks both the 1″ and 3″OH substituents, has greater flexibility in the S2-MacroD binding pocket (figure 3*c*). While the ADP-HPM pyrrolidine ring is overall similarly orientated to the distal ribose in the S2-MarcoD:ADPr structure, movement of the 2″OH group by 2.3 Å relative to its position in ADPr leads to ring relaxation, while only maintaining the Asn244 contact to the protein.

Interestingly, the S2-MacroD:ADP-HPD structure shows electron density for two additional ligand molecules outside the canonical binding sides, one in protomers A and one in protomer D (figure 3*d*,*e*). Each of these extra ligands is closely facing the corresponding canonical bound ADP-HPD molecule so that their C1″ atoms are in close proximity to the 2′OH moieties of the ADP-HPDs in the respective binding clefts (distance: 3.9 Å and 3.3 Å in protomer A and D, respectively; figure 3*f*,*g*).

During the preparation of this manuscript, several other S2-MacroD structures became available in the RCSB database [74–78]. We compared these with our S2-MacroD2 structures (i.e. crystallization conditions and crystal parameters) and found that crystals form under various different conditions, in several space groups (*P*1, *P*2_1_, *P*2_1_2_1_2_1_, *C*2, *P*4_1_ and *P*4_1_2_1_2), and diffracting to different resolution (reported from 0.95 to 2.6 Å). This can partially be explained by the inherent propensity of several macrodomains to form crystals in the presence or absence of distinct ligands in the crystallization conditions [79]. Close inspection of the higher-order arrangements within the crystals revealed in addition that small alterations in the N- and C-termini of the constructs appear to have a major influence on crystallization behaviour as they partake in the formation of crystal contacts. Structural superposition between our structure (PDB 6Z5T) and others bound to ADPr showed very high agreement, reflected in r.m.s.d. values ranging from 0.164 to 0.211 Å (PDB 6W02, 6WYL and 6WOJ; electronic supplementary material, table S5). Note that this change is exaggerated by comparison with available *apo* forms (r.m.s.d. values between 0.258 and 0.366 Å; PDB 6WEY, 6WEN and 6VXS; electronic supplementary material, table S5). This is due to a rearrangement of the catalytic loop (H249 to Val253), which adopts a more relaxed conformation and moves approximately 2 Å out of the active site. In addition, the absence of the distal ribose allows Phe336 to rotate by approximately 50° into the binding cleft. Similar conformational changes can also be observed in the available S2-MacroD:AMP complex structure (PDB 6W6Y). Interestingly, the comparison of all structures containing a bound adenosine moiety showed that the edge-to-face interaction with Phe360 can only be observed in four out of seven structures. This might indicate that the Phe360 has only a minor role in the ADPr coordination.

### 3.4. Probing the ligand pocket

While the catalytic mechanism of MacroD-like enzymes is not fully understood, current evidence suggests a substrate-assisted mechanism in which precise positioning of the distal ribose plays an important role [70–72]. This is consistent with our structural observations, which show a tight coordination network surrounding the distal ribose, fixing it in its position. This is further supported by earlier studies showing that asparagine-to-alanine mutation of the position isostructural to Asn244 in S2-MacroD abolishes catalytic activity, probably due to ribose miss-positioning [21,32,45]. A second important aspect of the mechanism is the presence of a substrate-coordinated, activated water molecule placed between the Gly252 analogous position and P*^α^*, which was first discovered in a bacterial MacroD-like enzyme [80]. Presumably this water molecule acts as a nucleophile in a S_N_2-like displacement reaction. As yet, no evidence for a similar water molecule has been reported for viral macrodomains and all available ADPr structures show the ligand in its β-anomeric form, which is thought to be incompatible with the coordination of a potential catalytic water molecule. Using the automodification of PARP14 WWE-CAT as readout, we analysed a variety of S2-MacroD variants carrying single amino acid substitutions to probe for ligand binding and catalytic function (figure 3*h*). Disruption in the distal ribose coordination by either N244D, G334V and F336L nearly abolished the activity of S2-MacroD. Note, that we chose the N244D mutation, which is thought to be less physico-chemically severe in comparison to alanine mutation. We tested the requirement of the catalytic water molecule by steric displacement via introduction of a small side chain at Gly252 (G252V). This mutation led to a reduction in catalytic activity, supporting the idea that a substrate-activated water molecule partakes in the reaction.

In line with our structural observations, mutation F360L has no influence on catalytic activity, indicating that the contribution of this residue to the overall ligand binding is negligible. Interestingly, mutation of Asp226, which coordinates the C6 amine of the adenosine ring, to an asparagine (D226N) had no negative effect on enzymatic activity, however, this activity was severely impaired by the D226V mutation. This suggests that Asp226 is one of the key residues for ADPr-like ligand coordination. S2-MacroD activity was tested against different ADPr-like substrates using PARP14 WWE-CAT automodified with β-NAD^+^ as well as its analogues biotin- or ε-NAD^+^ (figure 3*i*). The secondary amine at the C6 position within the biotin-ADPr modification is partially tolerated as substrate, whereas the ε-ADPr base is not. This finding further supports the importance of the interaction of Asp226 with the adenosine base.

Taken together, we have mapped the ADPr binding site of S2-MacroD and identified several features that are crucial for its catalytic activity. It is important to consider that viral challenges impose an evolutionary pressure, which often results in proteins adapting and counter-adapting at the host–virus interface [81]. Several antiviral PARPs, including PARP14, are under positive selection in this way, supporting the idea that they are part of this arms race [20,69]. This also suggests that different adaptations at the host–virus interface in various species can have nuanced functional outcomes. Moreover, even relatively small changes in the catalytic efficiency or target selectivity between viral macrodomains may result in differences in the cellular ADP-ribosylation signalling and ultimately lead to differences in the viral host responses (e.g. IFN or inflammatory response).

### 3.5. Phylogenetic analysis of the ligand-binding pocket

**Figure 4.**
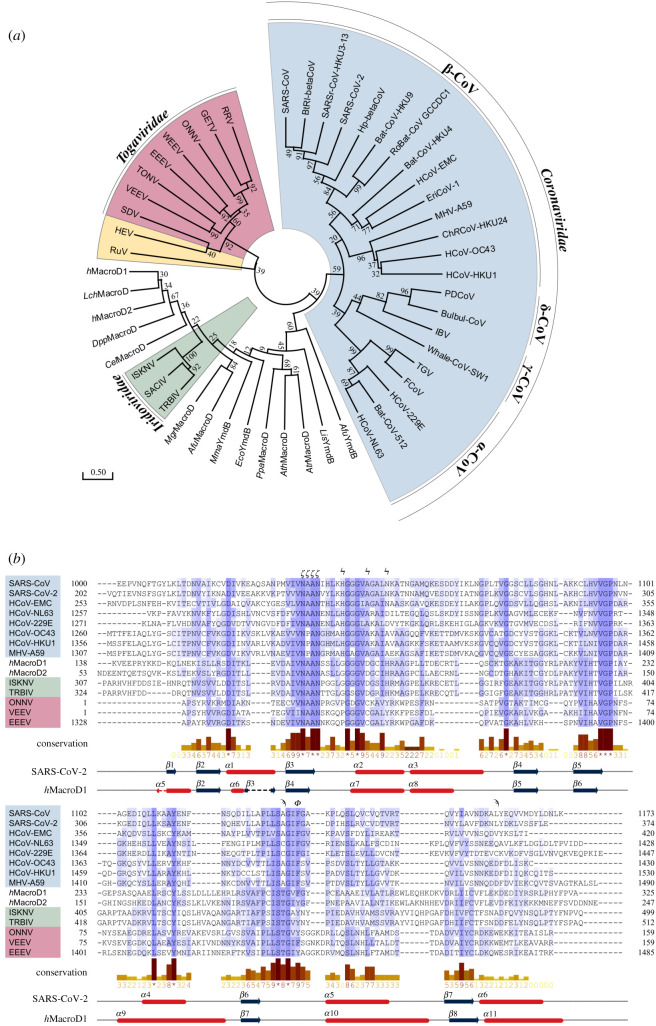
Phylogenetic analysis of MacroD-like domains. (*a*) Evolutionary phylogenetic tree analysis of MacroD-like domain: The tree was constructed with amino acid sequences isolated from their whole protein context by multiple sequence alignment. The evolutionary history was inferred using the maximum-likelihood method under the LG model of amino acid substitution as implemented in MEGA X. The tree with the highest log likelihood (−10 025.59) is shown. The tree is drawn to scale, with branch lengths measured in the number of substitutions per site and the percentage of trees in which the associated taxa clustered together is shown next to the branches. Clusters of viral macrodomains are highlighted (*Coronaviridae*, blue; *Iridoviridae*, green; *Togaviridae*, red; hepatitis E virus (HEV) and Rubella virus (RuV), yellow). The alignment used to construct the tree can be found in electronic supplementary material, figure S2 and sequence information in electronic supplementary material, table S1. (*b*) Multiple sequence alignment of representative MacroD domains. The secondary structure of S2-MacroD and *h*MacroD1 as well as the residue conservation of physico-chemical properties [85] are given underneath the alignment. Residues important for ligand binding and catalysis are indicated above the alignment: catalytic (ϟ), active site arene (ϕ), NAAN motif (ϛ) and proximal ribose binding (ϡ). Note, that the structurally resolved, but evolutionary not conserved, N-terminal region of *h*MacroD1 was omitted for clarity. Viral sequences are highlighted using the colour scheme described in (*a*).

To gain insights into the diversification of viral macrodomains and relate our structural findings to future drug development efforts, we performed a phylogenetic analysis of the ligand-binding pocket. One central aim was to identify features that are coronavirus specific so that viral macrodomains and the closely related human macrodomains *h*MacroD1 and 2 can be distinguished. Human disease-causing CoVs were found to be of zoonotic origin, with strains causing severe infections (MERS, SARS and SARS-CoV-2) associated with betacoronviruses with recent wildlife origin, while milder infections are caused by alpha- and betacoronaviruses (α-CoVs: HCoV-229E and HCoV-NL63; β-CoVs: HCoV-HKU1 and HCoV-OC43) that have adapted to the human host [82–84]. In order to analyse the specific features of S2-MacroD and probe commonalities and differences with other macrodomains, phylogenetic trees were constructed with sequences restricted to (i) betacornonaviruses, and (ii) spanning all domains of live as well as viruses (figure 4; electronic supplementary material, figures S1, S2 and table S1). For the viral sequences analysed, these domain-level phylogenies reproduce previously described whole-genome relationships [86–88] and show genera and linage specific branching. In this analysis, *Coronaviridae* and *Togaviridae* sequences form distinct clades with Rubella virus (RuV) and hepatitis E virus (HEV) located within the alphavirus clade. Macrodomains from the *Iridoviridae* form a sister group to the *Animalia* (figure 4*a*,*b*) and our analysis showed that macrodomain sequences are nearly exclusively limited to the *Megalocytivirus* genus, which may suggest that these macrodomains arose through recent lateral gene transfer from the host. Based on our protein phylogeny, we probed this hypothesis by constructing a tree of macrodomain encoding nucleotide sequences form *Iridoviridae*, their fish hosts, other *Animalia*, *Fungi* and *Plantae* (electronic supplementary material, figure S3 and table S3). In contrast to the amino acid sequences, *Iridoviridae* are not placed within the *Animalia* and *Fungi* clade, but form a sister group (electronic supplementary material, figure S3*a*). Reflecting the protein phylogeny, the *Iridoviridae* and *Animalia* are closer related than the *Iridoviridae* and *Plantae*. Together, this suggests that either no direct transfer occurred, the MacroD sequence was transferred from a so far unidentified species or that the MacroD sequence has undergone rapid diversification following the transfer. Using extensive BLAST searches we were unable to find coding sequences closely related to the *Iridoviridae* and further investigations are required to determine the origin of this MacroD branch.

**Figure 5.**
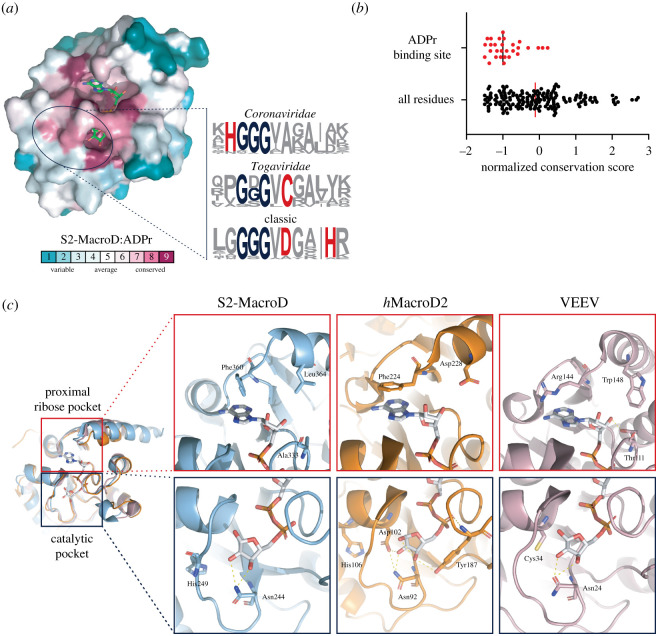
Evolutionary differences in the proximal ribose and catalytic pocket. (*a*) Surface representation of residue conservation analysis for the MacroD-like class carried out using ConSurf server and mapped onto the S2-MacroD:ADPr structure. Colouring represents continuous conservation scores partitioned into nine bins for visualization. Sequence frequency logos of the catalytic loop region were generated using WebLogo [89]. Proposed catalytic residues are highlighted in red and the triple-glycine motif in blue. (*b*) Scatter plot of normalized continuous conservation scores of (*a*) comparing residues in the ADPr binding site with all residues in S2-MacroD. Lower values represent higher degree of conservation. The median of the distribution is indicated. (*c*) Ribbon-liquorice representation of key residues in the proximal ribose and catalytic pocket for S2-MacroD (blue, PDB 6Z5T), *h*MacroD2 (orange, PDB 4IQY) and VEEV (old pink, PDB 3GQO). ADPr is given in white and polar interaction as dotted lines.

Next, we searched for evolutionary conserved motifs in the ADPr binding cleft. Overall, residues within the binding pocket showed a high degree of conservation (figures 4*b*, 5*a*,*b*). The adenosine coordination by Phe360 is, with the exception of nobecoviruses, unique to SARS-CoV-2 among betacoronaviruses (figure 5*c*; electronic supplementary material, figure S1), and hence is not found in SARS-CoV and MERS-CoV, which interact with the adenosine ring via an asparagine. However, other groups, including alphacoronaviruses, *Iridoviridae* and *h*MacroD1/2, possess a phenylalanine or tyrosine (Phe224 in *h*MacroD2) at this position (figure 5*c*; electronic supplementary material, figure S1). Interestingly, this may indicate that Phe360 is a recent (re)emerging feature, providing an explanation as to why it is the only known macrodomain demonstrating an edge-to-face interaction with the adenosine moiety. A further distinguishing feature is located four residues downstream of this phenylalanine in the primary sequence (figures 4*b* and 5*c*). This aspartate residue (Asp228 in *h*MacroD2) is conserved in *Iridoviridae* and *Animalia* which, in conjunction with Thr187 (*h*MacroD2) of the pyrophosphate binding loop, interacts with the proximal ribose via water-mediated contacts (figure 5*c*). Alphacoronaviruses have an isostructural glutamate (Glu1424 in HCoV-229E) instead of the aspartate, which directly interacts with the 2′ and 3′OH of the proximal ribose. In betacoronaviruses, the two residues are aliphatic (Ala333 and Leu364 in S2-MacroD), with the exception of embecoviruses, which have an aspartate instead of a leucine (figure 5*c*). In nearly all *Togaviridae*, these residues are replaced by a threonine and a tryptophan, respectively (figure 5*c*). Taken together these localized, macrodomain clade-specific variations result in distinct electrostatic microenvironments (electronic supplementary material, figure S4) as well as specific residue functionalities which may prove exploitable for drug design.

Further to the tight coordination described above, experimental data suggest that distinct variations in the catalytic mechanism have arisen within the MacroD-like class. This is supported by the identification of different catalytic residues in human and bacterial enzymes [70–72,80]. In humans, the conserved triple glycine is followed by a short α-helix containing a xDx(4)H motif (due to its first discovery and wide distribution, termed the ‘classic’ motif; figure 4*b*). This allows the histidine and aspartate to form a catalytic dyad, which interacts with the 2″OH moiety at the distal ribose, lowers the electron density at the sugar ring, and facilitates hydrolysis (figure 5*c*) [70–72]. This dyad is conserved among *Eukarya* and *Iridoviridae*, as well as in some bacteria, but not in other viruses (figure 5*a*; electronic supplementary material, figure S2). In *Togaviridae*, the catalytic aspartate is replaced by an absolutely conserved cysteine, which act as a nucleophile during the reaction cycle (figure 5*c*). This is significant as this would be a hitherto unrecognized catalytic residue. By contrast, CoVs have a non-functional alanine residue at the isostructural position. Additionally, close examination of this region revealed a highly conserved histidine residue (His249 in S2-MacroD) upstream of the triple glycine motif (figures 4*b* and 5*c*). This histidine makes side chain and water-mediated contacts both to the distal ribose as well as to both asparagines in the NAAN motif. This suggests that His249 is a key component for the correct positioning of the distal ribose in the catalytic cycle.

Together these findings suggest that at least three, conserved variations of a catalytic mechanism exist within the MacroD-like class: first, placement of the nucleophilic cysteine within the alphavirus macrodomain active site suggests either direct attack on the C2″ or trapping of a reaction intermediate, such as on oxocarbenium ion. During the preparation of this manuscript, new structures of the Getah virus (GETV) macrodomains became available showing a reaction between the conserved cysteine residue and the co-crystallized ADPr molecule (electronic supplementary material, figure S5) [90]. While the authors do not present direct evidence for its involvement in the catalytic mechanism, this finding lends further support to the idea of a catalytic role of this residue. Furthermore, both the prominent placement of the cysteine within the active site and its ability to react with ADPr makes it an interesting target for the development of covalent inhibitors. Second, *Eukarya* and *Iridoviridae* activate the substrate via a catalytic dyad, which could facilitate cleavage even in the absence of a bound catalytic water through formation of an oxocarbenium intermediate [71,72]. Finally, CoVs use the most radical form of a substrate assisted mechanism, relying primarily on the perfect positioning of the substrate distal ribose within the active site as well as requiring an activated water for an S_N_2-type attack on the C1″ position. Targeting the mechanism-specific interactions with small drug molecules that mimic the ligand interaction with this particular residue may offer specificity in term of inhibition.

**Figure 6.**
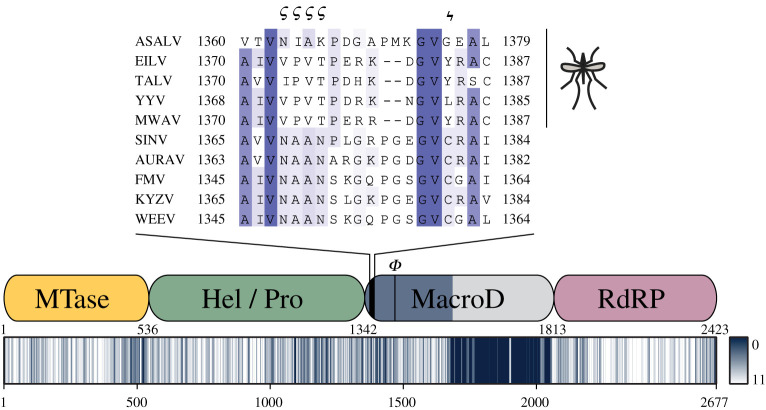
Loss of catalytic residues in the macrodomain of insect-restricted alphaviruses. Non-structural polyprotein (precursors of nsp1–4) sequences were aligned using Mafft L-INS-I and position-specific physico-chemical properties conservation scores plotted as heat map (lower panel; alignment position are indicated below the plot). Positions of nsp1–4 (nsp1, yellow; nsp2, green; nsp3 blue; nsp4, red) are indicated in the middle panel and inferred cleavage site positions for Agua Salud alphavirus (ASALV) given below the scheme. Names of the primary domains residing within these proteins are abbreviated as follows: MTase, guanine-7-methyltransferase (also possesses guanylyl-transferase [GTase] activity); Hel, helicase; Pro, protease; RdRP, RNA-dependent RNA polymerase. ϕ indicates the position of the aromatic residue important for distal ribose positioning in vertebra-infecting alphaviruses. This residue is substituted by lysine or glutamine in insect-restricted alphaviruses. The poor alignment and low conservation score in the C-terminal region of nsp3 (indicated in grey) is due to the presence of a hypervariable domain within this region. The alignment for the catalytic loop region of the macrodomain is given in the top panel and NAAN motif (ϛ) and proposed catalytic cysteine (ϟ) indicated above. The full alignment is given in electronic supplementary material, figure S4.

A striking insight into the importance of macrodomains comes from the recent emergence of a new, insect-specific group of alphaviruses, in addition to the known vertebra-infecting and aquatic alphaviruses. The host range of these viruses appears to be restricted to the former vectors, including mosquitos of the *Aedes*, *Anopheles* and *Culex* genera [91–95]. Sequence comparison of members of this group with closely related alphaviruses of the western equine encephalitis complex revealed specific loss of catalytically important residues within the macrodomain (figure 6; electronic supplementary material, figure S6). This correlation of loss-of-function mutations with the absence of antiviral PARPs from mosquito genomes (data not shown and [96]), lends evolutionary support to functional studies highlighting the importance of the macrodomains for the intracellular life cycle of viruses [39,40,97,98].

### 3.6. S2-MacroD as therapeutic target

The targeting of the viral macrodomain by future therapeutics could face two challenges: first, drugs can exert evolutionary pressure on the virus and force diversification to escape the negative impact of the compound on viral viability, and second because MacroD-like enzymes occur in all branches of life inhibitory compounds could cross-react not only with the host but also the host-associated microbiome. We assessed these problems by analysing the natural sequence variants of S2-MacroD and their underlying selection as well as investigating the human-associated microbiome for similarities with alpha- and coronaviral MacroDs.

Natural variants of S2-MacroD and their associated selection pattern were identified through the covid.galaxyproject [65] (table 1). The used analysis pipeline classifies selection based only on internal branches to mitigate the inflation of dN/dS values, but the resulting dataset may still contain positive selection attribution for sites not under such influence. A second inherent limitation is the immediacy of the pandemic: SARS-CoV-2 is a zoonotic pathogen and thus new variants may arise due to the ongoing adaptation to the human host. In the analysed data, we identified 25 residues with associated selection of which ten were classified as pervasive positive, two as episodic positive, and 13 as pervasive negative with six silent variants. We mapped all resulting variants to our S2-MacroD structure and analysed them for their potential influence on protein stability and enzymatic function. For the majority of sides (14), our analysis predicts no structural effect. As expected the predicted structurally disruptive variants are under negative selection, thus preserving protein integrity. The exception is Leu292, classified as pervasive positive, which occurs with low frequency as phenylalanine. Our analysis did not reveal any other macrodomain with this or a similar change. Three identified residues have the potential to influence ligand binding, Ala242 (negative), Ala243 (positive) and Ala333 (positive). All three have low-frequency valine variants. Ala242 and Ala243 are part of the conserved NAAN motif and as such their variants may affect distal ribose binding. While A242P variants can be found, e.g. in embecoviruses, Ala243 is absolutely conserved among all MacroD-like enzymes. This suggests that both variants are detrimental for enzymatic function. Together with the low occurrence frequency, this suggests that Ala243 may not be under true positive selection. Ala333 is situated close to the proximal ribose and the emerging change to valine would largely preserve the physico-chemical properties of the site. However, the increase of Van der Waals volume due to the valine sidechain may induce small changes in the local structure. Valines at isostructural positions can, for example, be found in the alphacoronaviruses Bat-CoV-512, FCoV and TGV (electronic supplementary material, figure S2 and table 1) and as such future diversification either natural or through drug-induced pressure appears feasible.

**Figure 7.**
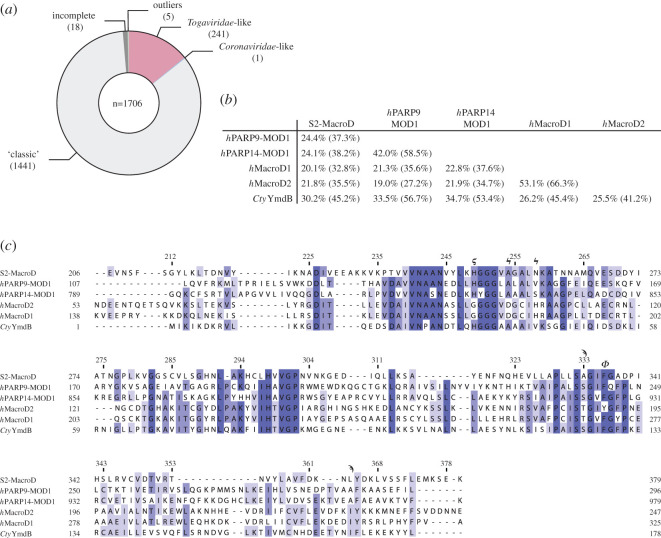
Assessment of similarities between viral, human and human-associated microbiome macrodomains. (*a*) The members of the human microbiome encode only a limited number of viral-like macrodomains. Sequences extracted from the MGnify database were aligned and classified according to the three identified catalytic motifs (compare figure 5*a*). Sequences denoted as incomplete lack either the aspartate or histidine from the ‘classic' motif and outliers have none of the catalytic residues. (*b*) Pairwise sequence identity comparison of S2-MacroD with closest human relatives (MOD1 of *h*PARP9 and 14 as well as *h*MacroD1 and 2) as well as the only identified *Coronaviridae*-like macrodomain from the human microbiome (*Clostridium tyrobutyricum* YmdB, *Cty*YmdB). Sequence identity and similarity (in parentheses) are provided. (*c*) Multiple sequence alignment of sequences analysed in (*b*). Important residues are indicated underneath the alignment: ‘classic' (ϟ) and *Coronaviridae* catalytic residues (ϛ), active site arene (ϕ) and residues involved in proximal ribose coordination (ϡ).

Next, we investigated possible cross-reactivity with other human macrodomains as well as the human-associated microbiome. Using the MGnify microbiome database [66], we identified 1706 sequences containing the catalytic region as identified in figure 5*a*. The majority of these sequences (1441) showed the ‘classic' MacroD motif, whereas 241 contained the potential catalytic cysteine found in *Togaviridae*, and only one sequence from *Clostridium tyrobutyricum* contained the coronaviral catalytic motif (figure 7*a*). Surprisingly, comparison of S2-MacroD with the human macrodomains showed that *h*MacroD1 and 2 are not the closest relatives, but rather macrodomain 1 of PARP9 and macrodomain 1 of PARP14 (figure 7*b*). Alignment of these four sequences with S2-MacroD showed the presence of the distinctive HGGG motif in MOD1 of PARP9 and PARP14 as well as the aromatic residue involved in distal ribose positioning (Phe360 in S2-MacroD) (figure 7*c*). However, none of the sequences showed similarities in the proximal ribose binding area, identified as macrodomain selective region. The sequences carrying the *Togaviridae*-specific cysteine are diverged in the proximal ribose binding region: in 89 sequences an alanine isostructural to Ala333 in coronavirus was identified, which in three sequences co-occurs with a leucine (like in S2-MacroD) and in 36 sequences with another aliphatic residue (Ala, Ile or Val). The *Togaviridae* proximal ribose residues combination (Ser/Thr paired with Trp) was only observed twice. Together this suggests that both alphavirus and SARS-like coronavirus macrodomains can be selectively targeted without a high likelihood of cross-reactivity with the human host or microbiome. This further underlines the suitability of viral macrodomains as therapeutic targets.

### 3.7. PAR degradation by viral macrodomains

Characterized MacrodD-like hydrolases from bacteria and humans have been described as mono(ADP-ribosyl)hydrolases [70], but it appears that some viral MacroD-like domains may have evolved a significant PAR degrading activity [33]. Specifically, the authors observed a dependence of the activity on the viral origin with macrodomain derived from HCoV-229E showing low, SARS-CoV intermittent and VEEV efficient PAR degradation. This is surprising as so far PAR chain degradation has been restricted to two other types of enzymes: PARG, an enzyme containing an evolutionary distinct macrodomain-fold, and the structurally unrelated (ADP-ribosyl)hydrolase 3 (ARH3) [70].

**Figure 8.**
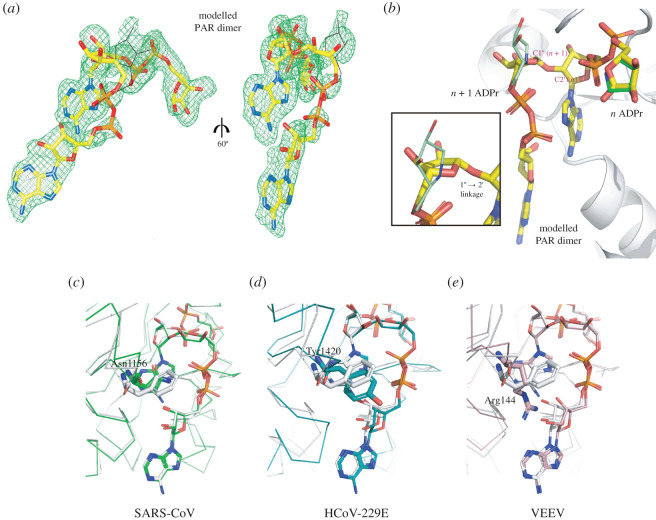
Two ADP-HPD ligands bound to S2-MacroD mimic PAR dimer binding. (*a*) Modelled PAR dimer (yellow sticks) placed in the composite omit map (countered at 2*σ*) of the two ligands ADP-HPD from S2-MacroD:ADP-HPD structure (orientation as figure 3*c*,*d*). Black lines trace the original position of the ADP-HPD ligand. (*b*) Close up of the overlay between the model PAR dimer (yellow) and the two ADP-HPD ligands (*n*, canonical ADP-HPD [dark green]; *n* + 1, non-canonical ADP-HPD [light green]) bound to S2-MacroD (white). The framed inset is a zoomed view of the distal ribose of the *n* + 1 ADPr highlighting the twist respect to the pyrrolidine ring of the second ADP-HPD ligand. (*c*–*e*) Energy minimized models of PAR dimer in selected viral macrodomains. The models were generated using (*c*) SARS-CoV:ADPr (PDB 2FAV), (*d*) HCoV-229E:ADPr (PDB 3EWR) and (*e*) VEEV:ADPr (PDB 3GQO) as initial structures and overlaid to S2-MacroD-PAR dimer model in white. Amino acids corresponding to Phe360 of S2-MacroD structure are shown in sticks and are Asn1156 (*c*), Tyr1420 (*d*) and Arg144 (*e*) with numbering corresponding to alignment in electronic supplementary material, figure S2.

Interestingly, our S2-MacroD:ADP-HPD structure showed the presence of an additional ADP-HPD molecule outside the canonical binding sites in protomers A and D. The joint positioning of the canonically and non-canonically bound ADP-HPD molecules closely resembles a PAR dimer conformation. The pyrrolidine C1″ of the molecule outside the binding site (*n* + 1 position) is positioned 3.3 Å away from the 2′OH of classically bound ADP-HPD (*n* position) and its orientation is compatible with the expected α-anomeric linkage (figure 3*f*). Furthermore, the *n* + 1 ADP-HPD is held in place by interactions by its C6 amine with Glu229 as well as by its 3″OH with the C6 amine of *n* (figure 3*f*). Guided by the clear electron density of the two bound ADP-HPD ligands, we modelled a PAR dimer into the S2-MacroD structure (figure 8*a*,*b*). The superposition of the energy minimized model with the S2-MarcoD:ADP-HPD structure revealed a near perfect agreement between the model and the two adjacent ADP-HPD molecules. The one exception was a small rearrangement of the *n* + 1 distal ribose to allow for the ribose(1″ → 2′)ribose bond formation (figure 8*b*). This arrangement of the PAR dimer in the S2-MacroD structure differs from earlier studies of the PARG:PAR dimer complex, which show PARG binding to the chain end (ADPr units in the *n* and *n* − 1 position) [99]. In this structure the *n* − 1 ADPr unit interacts only weakly with the PARG enzyme, indicating that canonically bound ADPr unit provides most of the binding energy. Furthermore, the position of the substrate within the structure is consistent with the observation that PARG is primarily an *exo*-glycohydrolase releasing ADPr units from the chain end [100]. The *n*/*n* + 1 positioning of the PAR dimer in the S2-MacroD model is more consistent with *endo*-cleavage or the release of PAR chains by cleavage of the protein-terminal ribose bond as observed for human TARG1 [101]. Therefore, this suggests that viral macrodomains either have less *endo*/*exo* selectivity or are able to generate free PAR. To gain further insights into the structural basis for the observed difference in PAR degradation activity between viral species, we generated energy minimized models of the HCoV-229E, SARS-CoV and VEEV macrodomains in complex with PAR dimers (figure 7*c*–*e*). Most strikingly, we found that the adenosine-coordinating residue is potentially the primary indicator for the ability to degrade PAR chains. Our HCoV-229E:PAR dimer model shows that Tyr1420 is displaced from adenosine coordination to allow positioning of the proximal ribose of the *n* + 1 ADPr (figure 8*d*). Furthermore, the new ribose(1″ → 2′)ribose linkage forces the 2′OH group of the *n* ADPr, involved in this bond, to rotate by approximately 2.4 Å out of the binding pocket. This disrupts the interaction of both the 2′ and 3′ OH group with Glu1424 further destabilizing the binding of the ADPr unit within the cleft. By contrast, from our models, the potential interaction of SARS-CoV and VEEV macrodomain with the *n* + 1 ADPr does not weaken the binding of the *n* ADPr unit in the binding cleft. Phe360 in S2-MacroD maintains its interaction with the adenosine moiety of the canonically bond ADPr and neither hinders nor contributes to the binding of the *n* + 1 ADPr (figure 8*c*). Asn1156 in the SARS-CoV and Arg144 in the VEEV macrodomain appear to further stabilize the PAR dimer complex through additional ligand interactions: both bind with the 2′ OH moiety of the proximal ribose of the *n* + 1 ADPr, while SARS-CoV Asn1156 further interacts with its P*^β^* phosphate and VEEV Arg144 with its adenosine ring (figure 8*e*). Together, these potential interactions and interpretations of our models correlate well with the experimental PAR degrading activities [33], and provide a valuable structural basis for further investigation. Whether PAR degrading activity of coronaviral macrodomains has a physiological function and whether it is linked to differential pathogenesis between different viruses remain open questions. For alphaviruses it was shown that both PAR binding and hydrolytic activity, two overlapping but distinct functions, are required for viral replication [97]. This invites speculation as to the physiological advantage PAR chain degradation or sequestration grants viruses. Nsp3 is anchored in the endoplasmic reticulum via two transmembrane regions with the macrodomain facing the cytoplasm (figure 1*b*). Cytoplasmic PAR is primarily limited to two sources: nuclear-derived unconjugated (free) PAR- [102,103] or PARP5a/b (also termed tankyrase 1 and 2)-derived. First, nuclear generation of free PAR chains occurs primarily through PARP1, which acts as a nuclear stress sensor. Hyperactivation of PARP1 can trigger cell death by activating apoptosis-inducing factor (AIF) through association of AIF with free PAR at the outer mitochondrial membrane [102]. Less severe stress induces a reversible response wherein nuclear-derived PAR associates with PARP12 at the trans-Golgi network, leading to the release of PARP12 from the membrane, the formation of stress granules, and blockage of anterograde-membrane trafficking [104]. ER-to-Golgi trafficking is required to complete the intracellular life cycle of both corona- and alphaviruses [105]. Therefore, it can be assumed that nuclear-derived PAR would contribute to the creation of an antiviral environment. Importantly, PAR-degradation/binding by viral macrodomains would prevent PARP12 activation and slow cell death, increasing the chances of successful viral replication (figure 1*a*). Alternatively, PARP5a/b are key regulators of Wnt signalling, which is emerging as a new mechanism by which the host immune response can be shaped following infection [106]. For example, it was shown that human cytomegalovirus (HCMV) suppresses PARP5a auto-PARylation, presumably in a hydrolase-independent manner, reducing Wnt signalling and aiding HCMV replication [107]. Similarly, viral PAR-degrading macrodomains could interfere with PARP5-dependent signalling and thus suppress the formation of an antiviral environment (figure 1*a*). While considering both forms of cellular PAR signalling, one should take into account that the ability of viral macrodomains to degrade PAR varies considerably between virus species [33]. Therefore, it presents an additional facet to the host–virus arms race and may result in different physiological responses. Investigating the consequences of a trade-off between mono- and poly-ADP-ribosylation reversal on viral infectivity and whether disease severity correlates with macrodomain-dependent PAR degradation are two exciting avenues for future studies.

Taken together, the results presented here provide evidence for a functional diversification within the MacroD-like class. Furthermore, we demonstrate for the first time direct degradation of PARP14-derived ADP-ribosylation by a viral macrodomain and established the basis for PAR degradation. These insights may be used in future studies aiming to elucidate the physiological substrates of antiviral PARPs, which so far remain largely unknown. Furthermore, the structural and functional features of viral macrodomains discussed provide directions for future inhibitor development.

## Supplementary Material

Supplemental Tables and Figures

## Data accessibility

Atomic coordinates and structure factors for the reported crystal structures have been deposited in the Protein Data Bank under accession numbers 6Z5T, 6Z6I and 6Z72. The accession numbers of all datasets used in this article as well as the resulting alignments have been uploaded as part of the electronic supplementary material.
