# Supplementary material for: Viral macrodomains: a structural and evolutionary assessment of the pharmacological potential
Source: Open Biol. 2020 Nov 18;10(11):200237. doi: 10.1098/rsob.200237 (PMC7729036; doi:10.1098/rsob.200237)
Supplement: Supplemental Tables and Figures [file rsob200237supp1.pdf]

SUPPLEMENTAL MATERIAL

Viral Macrodomains: A Structural and Evolutionary Assessment of the Pharmacological Potential

Johannes Gregor Matthias Rack[1], Valentina Zorzini[1], Zihan Zhu[1], Marion Schuller[1], Dragana Ahel[1], Ivan Ahel[1]

[1]Sir William Dunn School of Pathology, University of Oxford, South Parks Road, Oxford, OX1 3RE, UK

Corresponding authors:

Ivan Ahel
tel:(+44) 1865 285-656; email: ivan.ahel@path.ox.ac.uk

Johannes G. M. Rack
tel: (+44) 1865 285-655; email: johannes.rack@path.ox.ac.uk

Article d.o.i.: 10.1098/rsob.20160237

Table S1. Data Collection and Refinement Statistics.

PDB accession code	**S2-MacroD:ADPr**<br>6Z5T	**S2-MacroD:ADP-HPD**<br>6Z6I	**S2-MacroD:ADP-[illegible]**<br>6Z72
Data Collection			
Synchrotron/beamline	DLS/I03	DLS/I03	DLS/I03
Wavelength (Å)	0.976230	0.976230	0.976230
Space group	$P2_12_12_1$	$P2_1$	$P2_1$
a (Å)	58.78	59.81	59.81
b (Å)	76.33	83.09	83.13
c (Å)	82.32	84.36	84.28
α (°)	90	90	90
β (°)	90	94.50	94.41
γ (°)	90	90	90
Content of AU	2	4	4
Resolution (Å)[a]	47.84 – 1.57<br>(1.62 – 1.57)	59.11 – 2.00<br>(2.07 – 2.00)	48.45 – 2.30<br>(2.38 – 2.30)
R_{sym} (%)[a,b]	35.33 (98.49)	17.17 (82.04)	15.74 (79.28)
Mean I/σ(I)[a]	4.09 (0.38)	2.61 (0.84)	3.27 (0.99)
Completeness (%)[a]	98.98 (93.63)	99.49 (99.46)	99.0 (97.4)
Redundancy[a]	13.1 (13.0)	2.0 (1.9)	2.0 (2.0)
$CC_{1/2}$ (%)[a]	99.5 (70.0)	97.4 (55.5)	98.2 (63.9)
Wilson B factor (Å^2)	21.56	16.51	28.76
Total number of measured intensities	684138	109016	73292
Number of unique reflections[a]	52368 (5198)	55663 (5531)	36664 (3607)
Refinement			
R_{cryst} (%)[c]	21.1	23.4	21.9
R_{free} (%)[d]	25.0	27.9	26.2
r.m.s.d. bond lengths (Å)	0.010	0.011	0.011
r.m.s.d. bonds angles (º)	1.56	1.47	1.47
Ramachandran (%) favoured/allowed/outliers	99.13/0.87/0.0	97.66/2.34/0.0	98.1/1.9/0.0
Rotamers (%) favoured/allowed/outliers	98.59 /1.41/0.0	98.36/1.64/0.0	98.3/1.7/0.0
MolProbity score	1.33	1.48	1.29
Average B factor (Å^2)			
Protein	26.49	24.72	43.43
Water	39.25	32.30	39.43
ADPr	22.45		
ADP-HPD		23.70	
ADP-HPM			40.69
Other ligands (incl. Na^+, EDO, GOL, etc.)	34.39	39.73	61.28

(a) Data for the highest resolution shell are given in parentheses.
(b) R_{sym} = Σ|/-</>|/Σ/, where / is measured density for reflections with indices hkl.
(c) R_{cryst} = Σ||Fobs| - |Fcalc||/Σ|Fobs|.
(d) R_{free} has the same formula as R_{cryst}, except that calculation was made with the structure factors from the test set.

Table S2. Amino acid sequences for phylogenetic analysis.

species		abbreviation	accession number[a]
**Vira**			
Coronavirinae[b]			
α-CoV	Feline coronavirus	FCoV	AAY32595
α-CoV	Human coronavirus 229E	HCoV-229E	NP_073549
α-CoV	Human coronavirus NL63	HCoV-NL63	YP_003766
α-CoV	Scotophilus bat coronavirus 512	Bat-CoV-512	YP_001351683
α-CoV	Transmissible gastroenteritis virus	TGV	NP_058423
β-CoV	Bat coronavirus RaTG13	Bat-RaTG13	QHR63299
β-CoV	Bat Hp-betacoronavirus/Zhejiang2013	Hp-betaCoV	YP_009072438
β-CoV	Bat SARS coronavirus HKU3-13	SARSr-CoV-HKU3-13	ADE34822
β-CoV	Betacoronavirus HKU24	ChRCoV-HKU24	YP_009113022
β-CoV	Coronavirus BtRl-BetaCoV/SC2018	BtRl-betaCoV	QDF43814
β-CoV	Hedgehog coronavirus 1	EriCoV-1	QCC20712
β-CoV	Human coronavirus HKU1	HCoV-HKU1	ARU07575
β-CoV	Human coronavirus OC43	HCoV-OC43	AGT51736
β-CoV	Middle East respiratory syndrome-related coronavirus	HCoV-EMC	YP_009047215
β-CoV	Murine hepatitis virus (A59)	MHV-A59	NP_045298
β-CoV	Pangolin coronavirus	Pan-CoV	QIA48640
β-CoV	Rousettus bat coronavirus GCCDC1	RoBat-CoV GCCDC1	YP_009273004
β-CoV	Rousettus bat coronavirus HKU9	Bat-CoV-HKU9	YP_001039970
β-CoV	Severe acute respiratory syndrome coronavirus	SARS-CoV	NP_828849
β-CoV	Severe acute respiratory syndrome coronavirus 2	SARS-CoV-2	YP_009725299
β-CoV	Tylonycteris bat coronavirus HKU4	Bat-CoV-HKU4	YP_001039952
γ-CoV	Beluga whale coronavirus SW1	Whale-CoV-SW1	YP_001876435
γ-CoV	Infectious bronchitis virus	IBV	ADA83575
δ-CoV	Bulbul coronavirus HKU11-934	Bulbul-CoV	YP_002308478
δ-CoV	Porcine deltacoronavirus	PDCoV	AKC54441
Togaviridae			
i.r.a.[c]	Agua Salud alphavirus	ASALV	EV83785
	Aura Virus	AURAV	NP_632023
	Eastern equine encephalitis virus	EEEV	AHL83792
i.r.a.[c]	Eilat virus	EILV	YP_006732326
	Fort Morgan virus	FMV	YP_003324587
	Getah Virus	GETV	YP_164438
	Kyzylagach virus	KYZV	AHL27150
i.r.a.[c]	Mwinilunga alphavirus	MWAV	BBC45634
	O'nyong'nyong virus	ONNV	NP_740705
	Ross River virus	RRV	QFR08125
	Sindbis virus	SINV	NP_062888
	Sleeping disease virus	SDV	NP_740655
i.r.a.[c]	Taï Forest alphavirus	TALV	YP_009333615
	Tonate virus	TONV	YP_009509052
	Venezuelan equine encephalitis virus	VEEV	NP_740698
	Western equine encephalitis virus	WEEV	NP_818935
i.r.a.[c]	Yada yada virus	YYV	QGR15362
Iridoviridae			
	Infectious spleen and kidney necrosis virus	ISKNV	NP_612244
	South American cichlid iridovirus	SACIV	AVR29678
	Turbot reddish body iridovirus	TRBIV	ADE34368
Others			
	Hepatitis E virus	HEV	AHC70111
	Rubella virus	RuV	NP_062883
Animalia			
	Caenorhabditis elegans	*Cel*MacroD	NP_502127
	Danaus plexippus plexippus	*Dpp*MacroD	XP_032524618

Homo sapiens	*h*MacroD1	NP_054786
Homo sapiens	*h*MacroD2	NP_542407
Homo sapiens	*h*PARP9	NP_001139574
Homo sapiens	*h*PARP14	NP_060024
Latimeria chalumnae	*Lch*MacroD	XP_005988903
Plantae		
Amborella trichopoda	*Atr*MacroD	XP_011622649
Arabidopsis thaliana	*Ath*MacroD	NP_030605
Physcomitrella patens	*Ppa*MacroD	PNR55811
Fungi		
Aspergillus fumigatus	*Afu*MacroD	XP_754239
Magnaporthe grisea	*Mgr*MacroD	XP_003720217
Monera		
Archaeoglobus fulgidus	*Afu*YmdB	WP_048064404
Clostridium tyrobutyricum	*Cty*YmdB	WP_017751878
Escherichia coli	*Eco*YmdB	WP_001460752
Legionella israelensis	*Lis*YmdB	WP_058501134
Magnetospirillum magneticum	*Mma*YmdB	WP_011384485

(*a*) GenBank accession number
(*b*) Abbreviation of corresponding *Coronavirinae* genus given before the species name: α-CoV, alphacoronavirus; β-CoV, betacoronavirus, γ-CoV, gammacoronavirus; δ-CoV, deltacoronavirus
(*c*) i.r.a., insect-restricted alphavirus

Table S3. Nucleotide sequences for phylogenetic analysis.

species	abbreviation	accession number[a]
**Vira**		
Iridoviridae		
Angelfish iridovirus	AFIV	MK689685
Banggai cardinalfish iridovirus	BCIV	MN432490
Giant seaperch iridovirus	GSPIV	KT804738
Infectious spleen and kidney necrosis virus	ISKNV	NC_003494
Orange-spotted grouper iridovirus	OSGIV	AY894343
Pompano iridovirus	PIV	MK098185
Red sea bream iridovirus	RSIV	AF506370
Rock bream iridovirus	RBIV	AY628698
Sea perch iridovirus	SPIV	AY628699
South American cichlid iridovirus	SACIV	MG570131
Turbot reddish body iridovirus	TRBIV	GQ273492
Animalia		
Caenorhabditis elegans	*Cel*MacroD	NM_069726
Danaus plexippus plexippus	*Dpp*MacroD	XM_032668727
Epinephelus lanceolatus[b]	*Ela*MacroD	XM_033630860
Etheostoma spectabile	*Esp*MacroD	XM_032528280
Homo sapiens	*h*MacroD1	NM_014067
Homo sapiens	*h*MacroD2	NM_080676
Lates calcarifer[c]	*Lca*MacroD	XM_018660235
Latimeria chalumnae	*Lch*MacroD	XM_005988841
Perca flavescens	*Pfla*MacroD	XM_028588884
Perca fluviatilis	*Pflu*MacroD	VHII01000010
Sander lucioperca[c]	*Slu*MacroD	XM_031303027
Scophthalmus maximus[c]	*Sma*MacroD	XM_035650424
Seriola dumerili[b]	*Sdu*MacroD	XM_022760297
Seriola lalandi dorsalis[b]	*Sld*MacroD	XM_023407344
Fungi		
Aspergillus fumigatus	*Afu*MacroD	XM_749146
Fusarium oxysporum f.sp. cubense RACE 1	*Foc1*MacroD	KB730000
Magnaporthe grisea	*Mgr*MacroD	XM_003720169
Plantae		
Amborella trichopoda	*Atr*MacroD	XM_011624347
Arabidopsis thaliana	*Ath*MacroD	NM_129622
Physcomitrium patens	*Ppa*MacroD	ABEU02000004

(*a*) GenBank accession number
(b) genus susceptible to *iridoviridae* infection
(c) confirmed *iridoviridae* host

Table S4. Pairwise sequence identity comparison of betacoronaviruses.

	HCoV-229E	MHV-A59	SARS-CoV	SARS-CoV-2	BtRI-betaCoV	SARSr-CoV-HKU3-13	Hp-beta-CoV	Bat-CoV-HKU9	RoBat-CoV GCCDC1	Bat-CoV-HKU4	HCoV-EMC	EriCoV-1	ChRCoV-HKU24	HCoV-OC43	HCoV-HKU1	Bat-CoV-RaTG13
MHV-A59	28.3% (41.9%)															
SARS-CoV	30.9% (45.7%)	29.0% (43.5%)														
SARS-CoV-2	28.9% (40.1%)	27.7% (44.6%)	70.6% (81.9%)													
BtRI-betaCoV	41.4% (59.7%)	27.9% (43.7%)	85.1% (86.2%)	68.1% (76.8%)												
SARSr-CoV-HKU3-13	32.3% (45.8%)	29.1% (44.7%)	78.2% (86.0%)	64.2% (74.1%)	74.4% (81.4%)											
Hp-beta-CoV	30.9% (43.5%)	32.3% (48.1%)	47.3% (62.4%)	47.6% (59.4%)	45.7% (58.4%)	45.9% (62.8%)										
Bat-CoV-HKU9	25.9% (40.1%)	29.2% (42.6%)	48.6% (62.9%)	46.6% (63.1%)	44.7% (58.0%)	42.3% (54.6%)	44.2% (63.0%)									
RoBat-CoV GCCDC1	27.2% (39.1%)	29.4% (44.8%)	45.5% (60.8%)	44.6% (59.9%)	41.5% (55.9%)	42.3% (55.2%)	37.2% (55.9%)	62.6% (72.1%)								
Bat-CoV-HKU4	24.6% (40.2%)	34.4% (51.1%)	39.2% (56.9%)	38.9% (56.7%)	36.3% (52.6%)	37.2% (55.1%)	43.2% (58.4%)	43.0% (54.2%)	40.6% (55.6%)							
HCoV-EMC	29.5% (45.1%)	36.5% (50.8%)	42.6% (61.9%)	40.0% (58.3%)	41.4% (59.7%)	39.4% (56.5%)	38.0% (53.5%)	41.5% (51.9%)	39.4% (50.0%)	57.1% (68.4%)						
EriCoV-1	25.7% (39.6%)	28.3% (43.4%)	39.3% (55.7%)	37.0% (55.0%)	38.0% (53.4%)	36.0% (55.5%)	35.4% (48.1%)	40.1% (55.0%)	39.1% (54.0%)	48.0% (58.5%)	57.5% (67.5%)					
ChRCoV-HKU24	25.0% (36.8%)	67.9% (79.1%)	31.4% (45.2%)	30.7% (46.0%)	28.0% (41.5%)	27.4% (43.8%)	27.5% (42.5%)	33.7% (47.4%)	26.6% (41.4%)	35.3% (50.8%)	37.5% (50.5%)	31.7% (45.7%)				
HCoV-OC43	26.8% (39.9%)	68.5% (77.2%)	30.3% (47.2%)	32.6% (49.7%)	30.4% (47.3%)	27.7% (43.6%)	31.6% (45.8%)	31.9% (48.6%)	30.5% (46.8%)	34.1% (52.0%)	39.8% (54.5%)	30.9% (44.9%)	72.2% (78.1%)			
HCoV-HKU1	28.9% (43.3%)	70.1% (79.3%)	31.7% (45.4%)	31.3% (49.7%)	31.4% (44.7%)	30.8% (44.6%)	31.4% (44.1%)	29.1% (42.2%)	29.4% (46.0%)	35.7% (48.9%)	37.8% (53.3%)	30.0% (44.9%)	66.3% (79.1%)	76.6% (86.9%)		
Bat-CoV-RaTG13	31.4% (43.8%)	27.7% (44.5%)	70.1% (81.0%)	93.6% (96.5%)	65.9% (75.1%)	63.7% (73.1%)	49.2% (60.1%)	47.1% (64.0%)	44.7% (59.8%)	39.8% (56.8%)	39.3% (59.0%)	35.0% (53.0%)	29.1% (45.0%)	32.0% (49.1%)	31.3% (48.6%)	
Pan-CoV	29.3% (45.0%)	25.3% (39.9%)	72.4% (83.9%)	79.8% (90.2%)	68.6% (77.3%)	63.7% (75.6%)	47.5% (59.6%)	45.7% (61.7%)	44.6% (59.4%)	41.5% (55.1%)	42.8% (59.5%)	36.8% (52.2%)	27.5% (41.0%)	30.3% (43.8%)	30.7% (46.9%)	79.9% (91.1%)

Table S5. Structural comparison between S2-MacroD and other viral macrodomains.[a]

genus	species	ligand	r.m.s.d.	# of Cα	PDB accession number	Ref.
α-CoV	FCoV	apo	0.872	92	3EW5	[1]
α-CoV	FCoV	ADPr	0.840	92	3JZT[b]	[1]
α-CoV	HCoV-229E	apo	0.836	98	3EWQ	[2]
α-CoV	HCoV-229E	ADPr	0.771	96	3EWR	[2]
β-CoV	Bat-CoV-HKU4	ADPr	0.602	129	6MEA	[3]
β-CoV	HCoV-EMC	apo	0.634	133	5HIH	[4]
β-CoV	HCoV-EMC	ADPr	0.577	130	5DUS	[5]
β-CoV	SARS-CoV	apo	0.352	121	2ACF	[6]
β-CoV	SARS-CoV	ADPr	0.258	136	2FAV[b]	[7]
β-CoV	SARS-CoV-2	apo	0.366	136	6WEY	[8]
β-CoV	SARS-CoV-2	apo	0.258	127	6WEN	[9]
β-CoV	SARS-CoV-2	apo	0.325	128	6VXS	[9]
β-CoV	SARS-CoV-2	ADP-HPD	0.156	152	6Z6I	This work
β-CoV	SARS-CoV-2	ADP-HPM	0.153	145	6Z72	This work
β-CoV	SARS-CoV-2	ADPr	0.211	132	6W02	[9]
β-CoV	SARS-CoV-2	ADPr	0.203	141	6YWL	[10]
β-CoV	SARS-CoV-2	ADPr	0.164	154	6WOJ	[11]
β-CoV	SARS-CoV-2	AMP	0.251	133	6W6Y	[9]
γ-CoV	IBV	apo	3.770	101	3EWO	[2]
γ-CoV	IBV	ADPr	3.487	98	3EWP	[2]

(a) If not indicated differently, values given are for comparison of S2-MacroD:ADPr protomer A with protomer A from the indicated PDB.
(b) Comparison with protomer B

FIGURE LEDGENS

Figure S1. Phylogenetic analysis of betacoronaviral macrodomains.

(*a*) Evolutionary phylogenetic tree analysis of MacroD-like domain from betacoronaviruses: the tree was constructed with amino acid sequences isolated from their whole protein context by multiple sequence alignment. The evolutionary history was inferred using the Maximum Likelihood method under the LG model of amino acid substitution as implemented in MEGA X. The tree with the highest log likelihood (-3783.65) is shown. The tree is drawn to scale, with branch lengths measured in the number of substitutions per site. Alignment used for this analysis is shown in (*d*). The different betacoronaviral lineages are highlighted in colour.

(*b*) Scatter plot of normalised continuous conservation scores of (*c*) comparing residues in the ADPr binding site with all residues in S2-MacroD. Lower values represent higher degree of conservation. The median of the distribution is indicated.

(*c*) Surface representation of residue conservation analysis for betacoronavirus macrodomains of the MacroD-like class carried out using ConSurf server and mapped onto the S2-MacroD:ADPr structure. Colouring represents continuous conservation scores partitioned into nine bins for visualisation.

(*d*) Multiple sequence alignment of representative betacoronaviral MacroD domains. The secondary structure of S2-MacroD as well as the residue conservation of physicochemical properties [12] are given underneath the alignment. Betacoronaviral lineages are highlighted using the colour scheme of (*a*). Sequence position relating to S2-MacroD are indicated above the alignment. Important residues are indicated above the alignment: catalytic histidine (ϟ) and NAAN motif (ς), active site arene (ɸ), and residues involved in proximal ribose coordination (ϡ).

Figure S2. Multiple sequence alignment of MacroD-class domains.

Multiple sequence alignment of representative all MacroD domains sequences used in the phylogenetic analysis in figure 4*a*. The secondary structure of S2-MacroD as well as the residue conservation of physicochemical properties [12] are given underneath the alignment. Sequence position relating to S2-MacroD are indicated above the alignment. Important residues are indicated above the alignment: catalytic residues (ϟ) and NAAN motif (ς), active site arene (ɸ), and residues involved in proximal ribose coordination (ϡ). Viral genera are highlighted using the colour scheme of figure 4*a*.

Figure S3. Analysis of the evolutionary origin of the *Iridoviridae* macrodomain.

(*a*) The evolutionary history of *Iridoviridae* macrodomains in relationship to *Animalia*, *Fungi*, and *Plantae* was inferred by using the Maximum Likelihood method and General Time Reversible model [13]. The tree with the highest log likelihood (-7431.83) is shown. The percentage of trees in which the associated taxa clustered together is shown next to the branches. The initial tree for the heuristic search were obtained automatically by applying the Maximum Parsimony method. A discrete Gamma distribution was used to model evolutionary rate differences among sites (5 categories (+*G*, parameter = 1.0627)). The rate variation model allowed for some sites to be evolutionarily invariable ([+*I*], 11.08% sites). The tree is drawn to scale, with branch lengths measured in the number of substitutions per site. Species identified as *Iridoviridae* hosts or residing in genera with known hosts are highlighted in red.

(*b*) Multiple sequence alignment of macrodomain coding sequences from *Iridoviridae* and their fish hosts. Sequence positions are related to ISKNV and indicated above the alignment.

Figure S4. Electrostatic surface potential analysis of selected macrodomains.

(*a*) Electrostatic surface potentials of S2-MacroD (PDB 6Z5T), *h*MacroD2 (PDB 4IQY), and VEEV macrodomain (PDB 3GQO) were calculated using APBS [14] integrated in PyMOL v2.3 (Schrödinger, LLC) with the non-linear Poisson-Boltzmann equation contoured at 5 kT/e. Negatively and positively charged surface areas are coloured in red and blue, respectively.

(*b*) Close up of the electrostatic surface potential shown in (*a*) for the ligand binding site.

Figure S5. The alphavirus-specific cysteine can form a covalent bond with ADPr.

Ribbon diagram structure of the Getah virus (GETV) macrodomain in complex with ADPr (pink; PDB 6R0R). The position of ADPr crystallised in complex with the VEEV macrodomain (silver; PDB 3GQO) is given for comparison. In GETV, the C1'' of ADPr is covalently linked to the highly conserved active site cysteine (Cys142).

Figure S6. Multiple sequence alignment of vertebra-infecting and insect-restricted alphaviruses.

Multiple sequence alignment of non-structural polyprotein of insect-restricted and closely related alphaviruses of the western equine encephalitis complex. Regions of nsp1-4 are indicated above the alignment using the colour scheme of figure 6. Important residues are indicated above the alignment: catalytic residues (ϟ) and NAAN motif (ς), active site arene (ϕ), and residues involved in proximal ribose coordination (ϡ). Sequence position is given in reference to ASALV above the alignment.

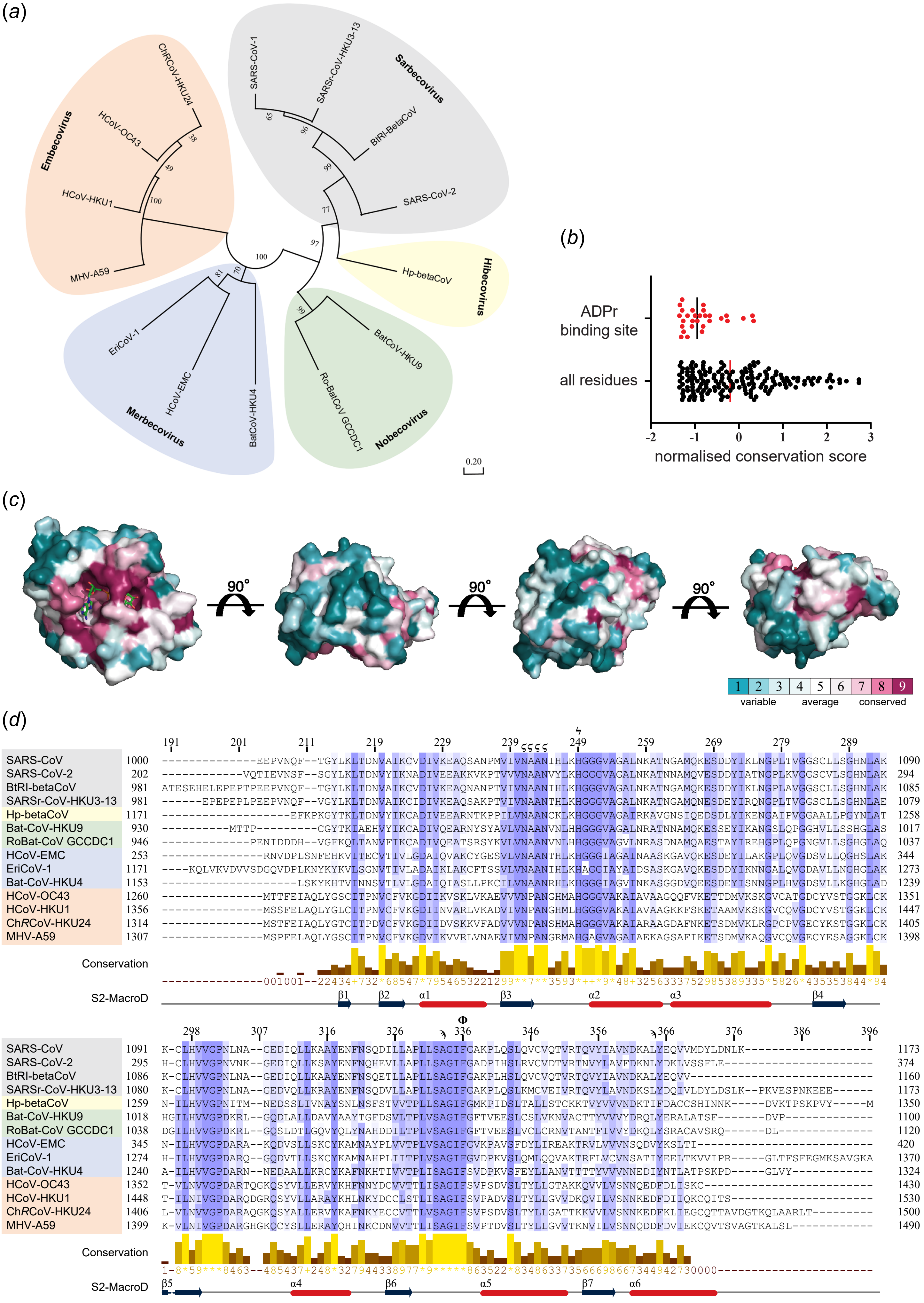
(a)
Embecovirus
ChRCoV-HKU24
HCoV-OC43
HCoV-HKU1
MHV-A59
Sarbecovirus
SARS-CoV-1
SARSr-CoV-HKU3-13
BtRl-BetaCoV
SARS-CoV-2
Hibecovirus
Hp-betaCoV
Nobecovirus
BatCoV-HKU9
Ro-BatCoV GCCDC1
Merbecovirus
EriCoV-1
HCoV-EMC
BatCoV-HKU4
0.20
(b)
ADPr binding site
all residues
normalised conservation score
(c)
90°
variable
average
conserved
(d)
SARS-CoV
SARS-CoV-2
BtRl-betaCoV
SARSr-CoV-HKU3-13
Hp-betaCoV
Bat-CoV-HKU9
RoBat-CoV GCCDC1
HCoV-EMC
EriCoV-1
Bat-CoV-HKU4
HCoV-OC43
HCoV-HKU1
ChRCoV-HKU24
MHV-A59
Conservation
S2-MacroD

Figure S1

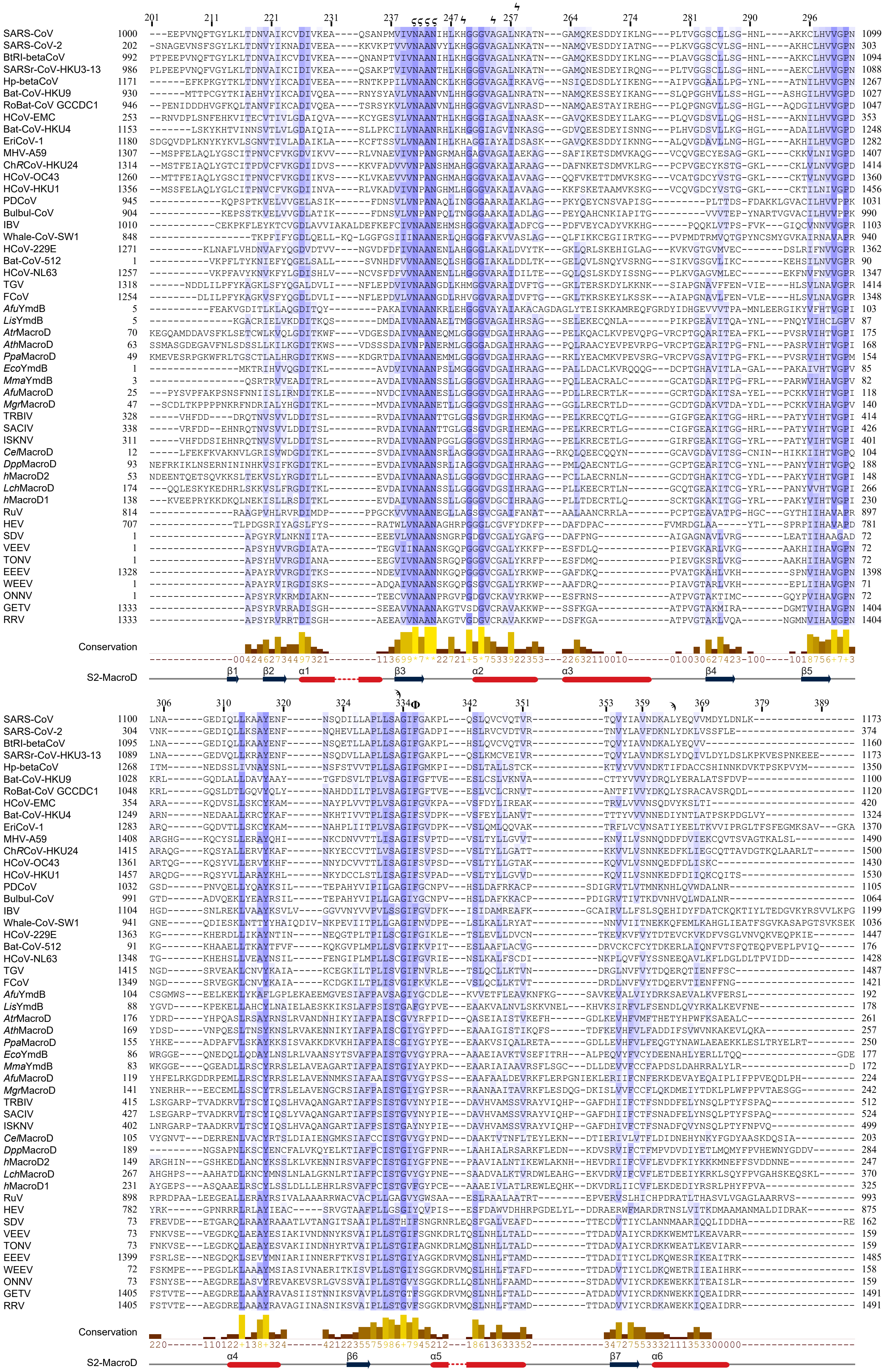
SARS-CoV
SARS-CoV-2
BtRl-betaCoV
SARSr-CoV-HKU3-13
Hp-betaCoV
Bat-CoV-HKU9
RoBat-CoV GCCDC1
HCoV-EMC
Bat-CoV-HKU4
EriCoV-1
MHV-A59
ChRCoV-HKU24
HCoV-OC43
HCoV-HKU1
PDCoV
Bulbul-CoV
IBV
Whale-CoV-SW1
HCoV-229E
Bat-CoV-512
HCoV-NL63
TGV
FCoV
AfuYmdB
LisYmdB
AtrMacroD
AthMacroD
PpaMacroD
EcoYmdB
MmaYmdB
AfuMacroD
MgrMacroD
TRBIV
SACIV
ISKNV
CelMacroD
DppMacroD
hMacroD2
LchMacroD
hMacroD1
RuV
HEV
SDV
VEEV
TONV
EEEV
WEEV
ONNV
GETV
RRV
Conservation
S2-MacroD
β1
β2
α1
β3
α2
α3
β4
β5
α4
β6
α5
β7
α6

Figure S2

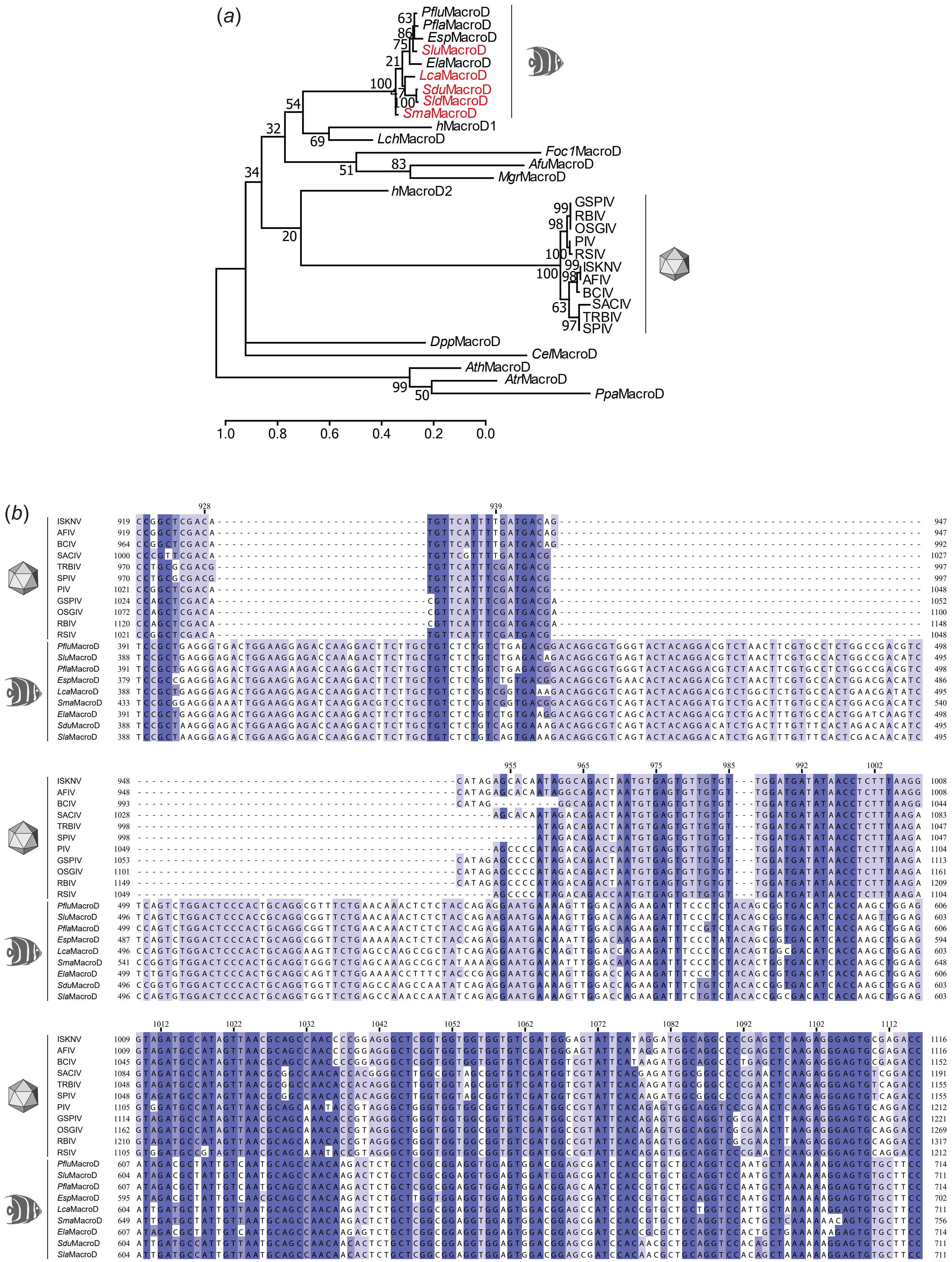

Figure S3 (page 1/2)

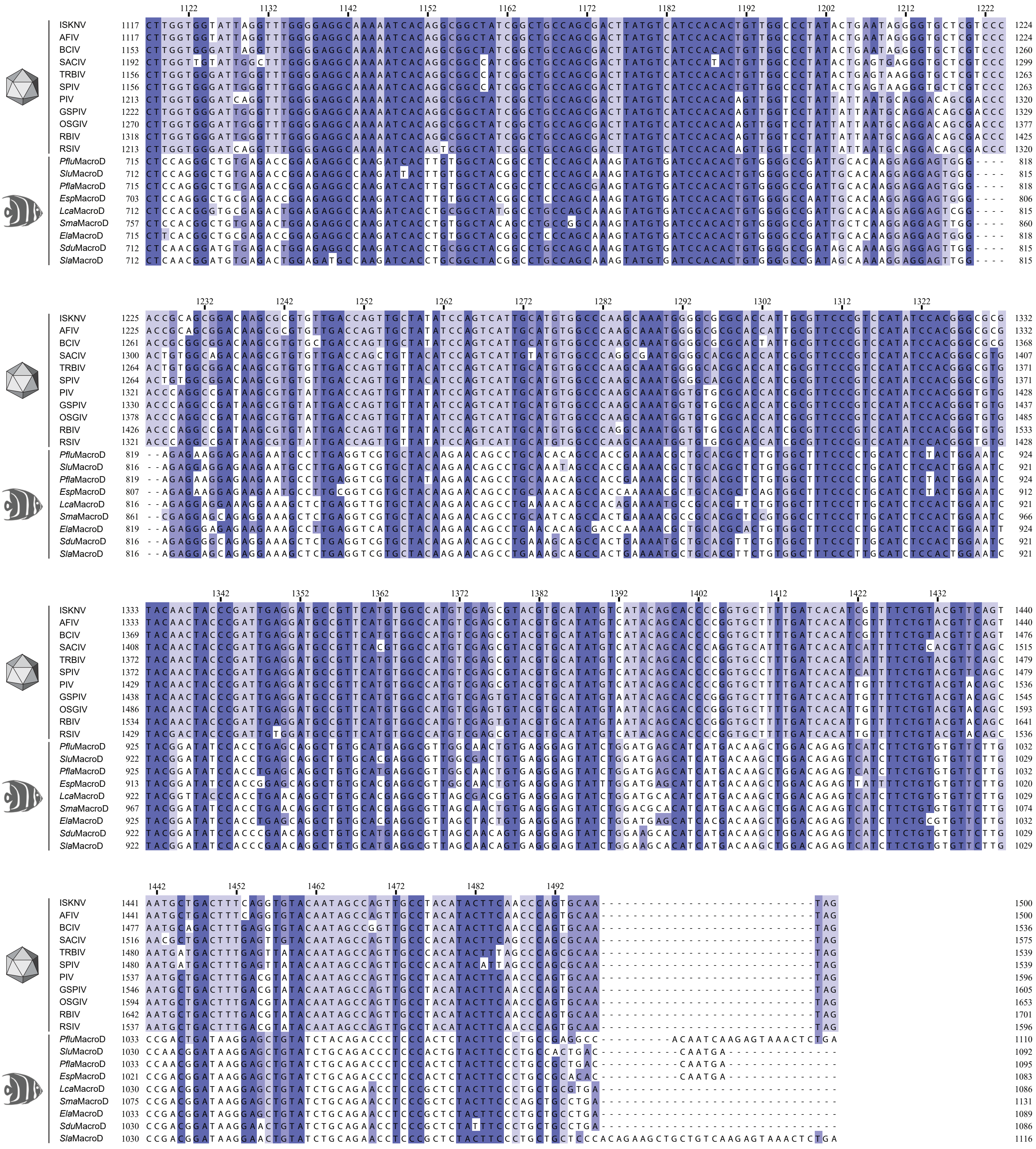

Figure S3 (page 2/2)

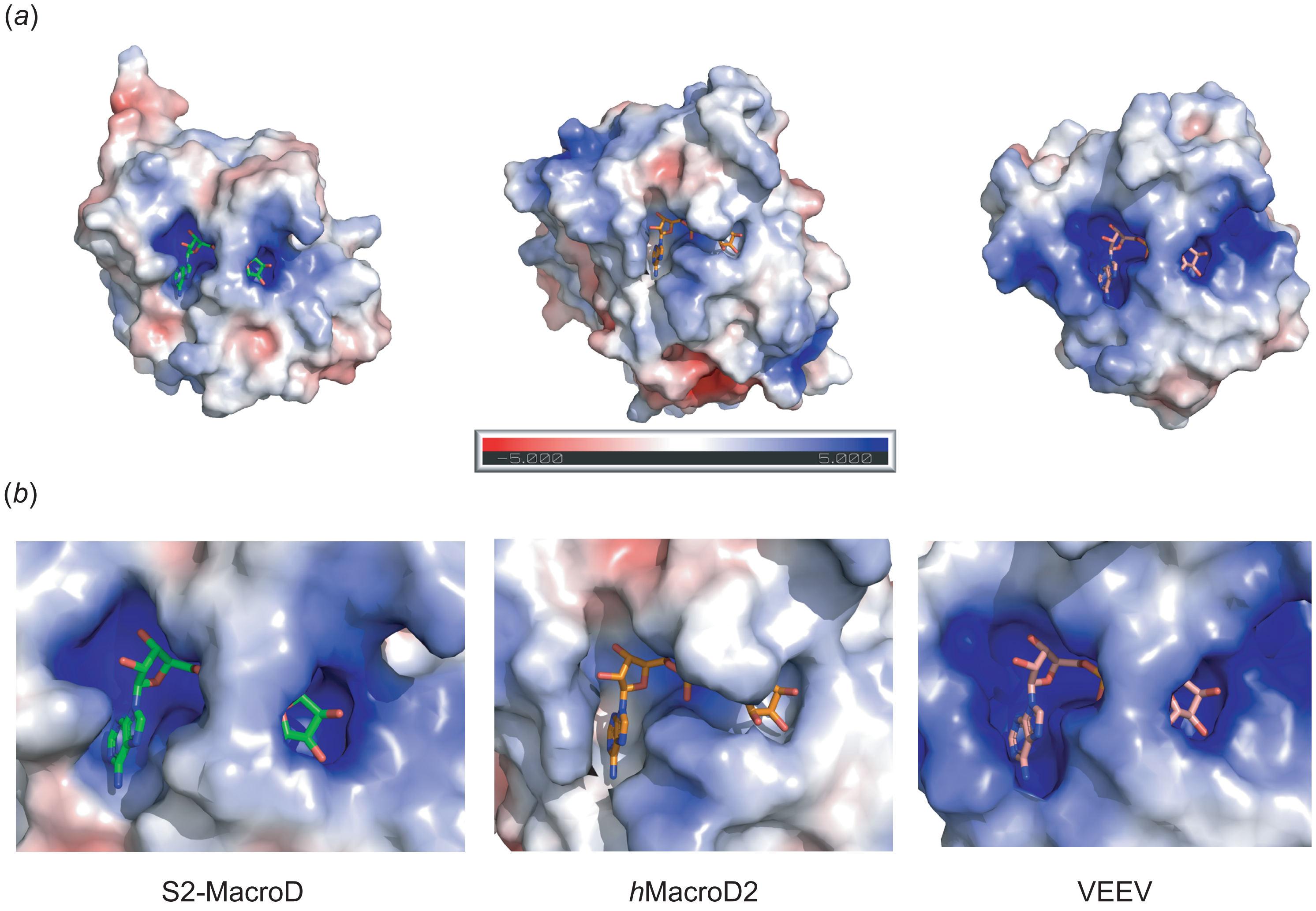

Figure S4

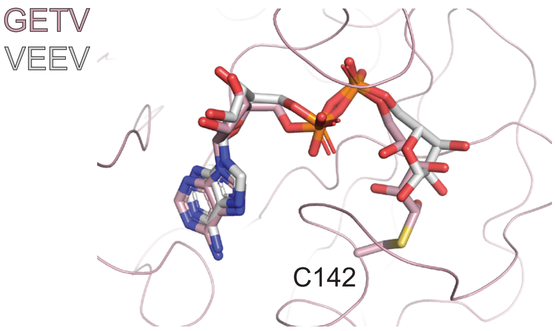

Figure S5

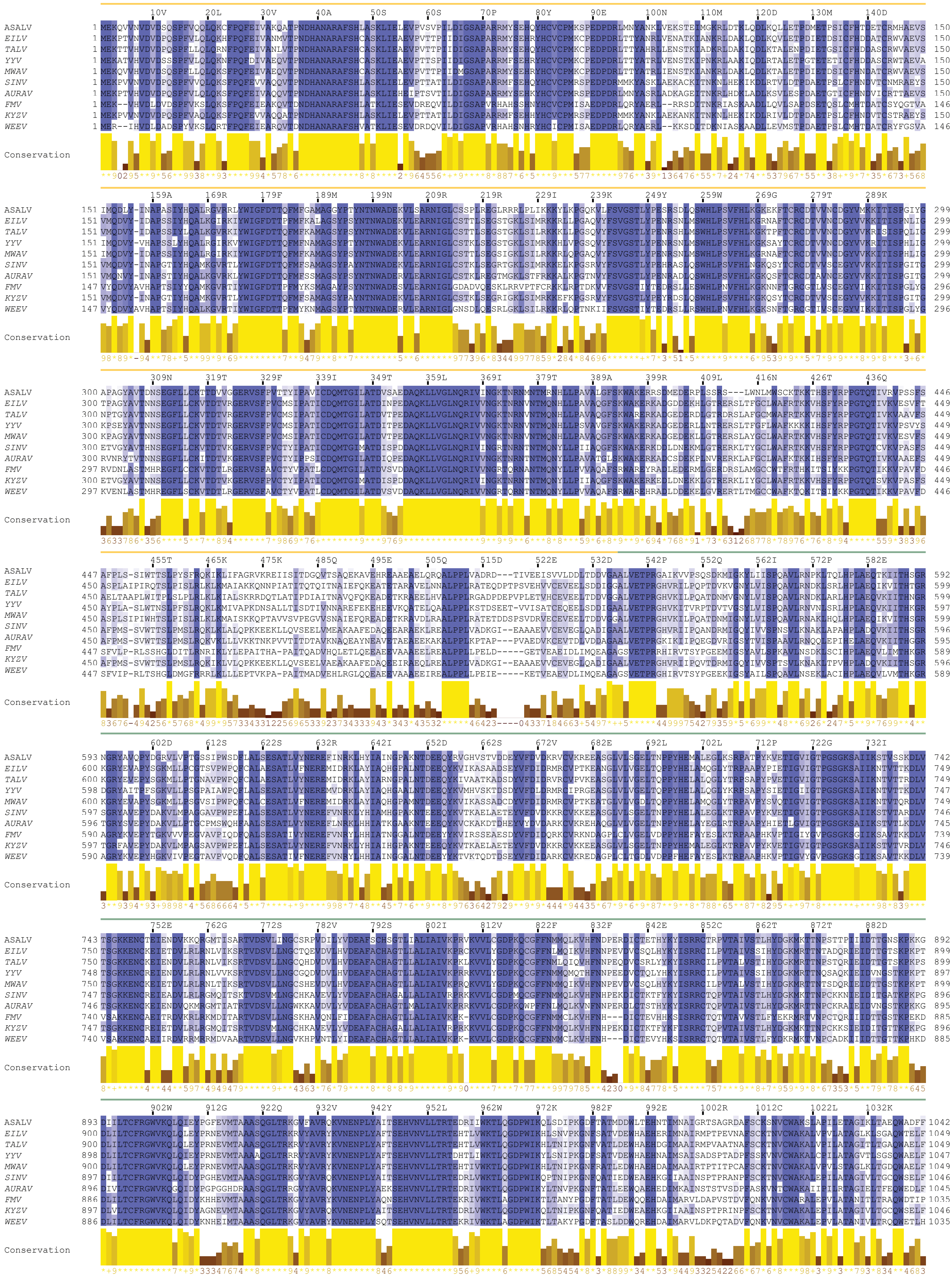

Figure S6 (page 1/3)

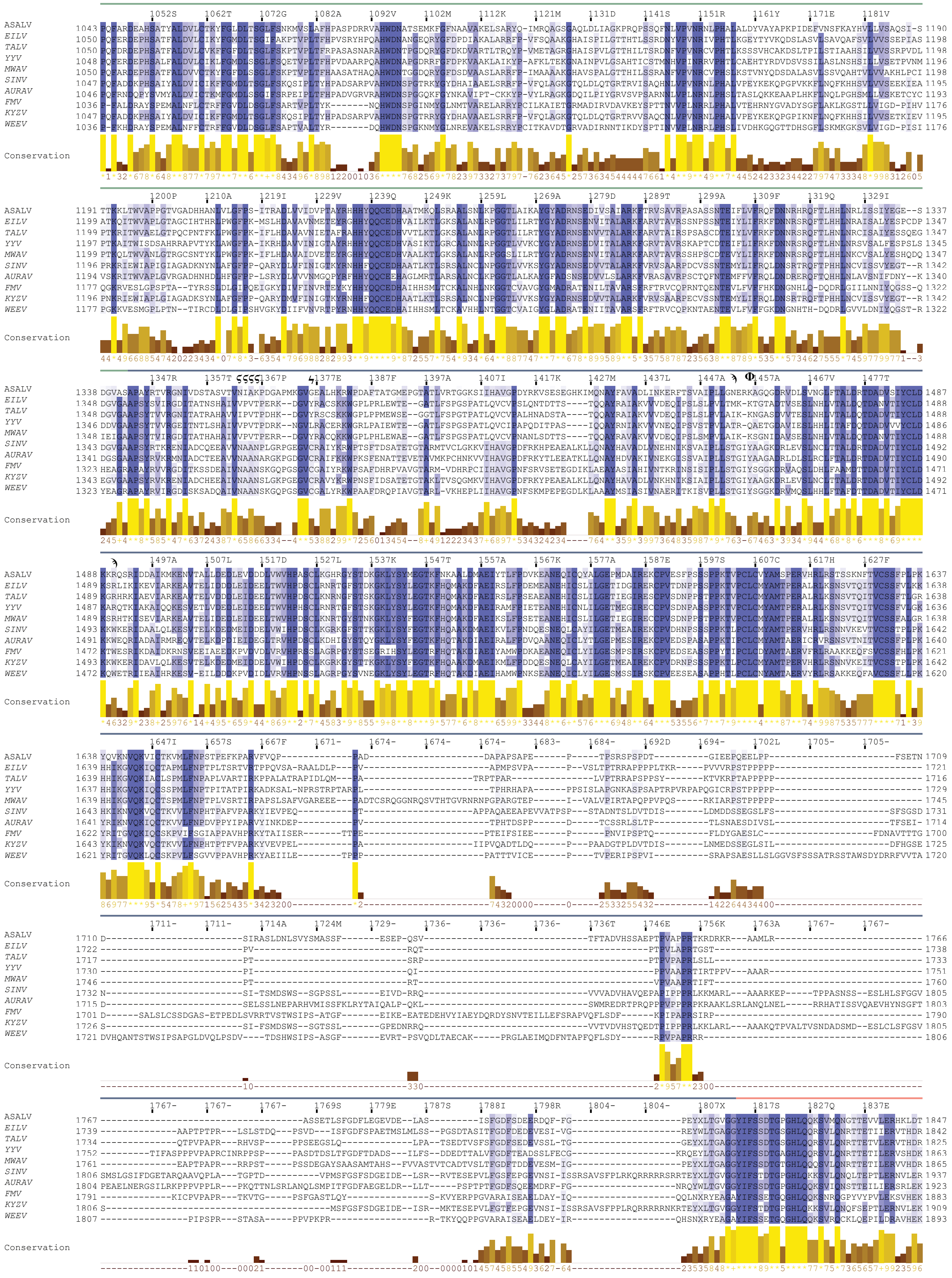

Figure S6 (page 2/3)

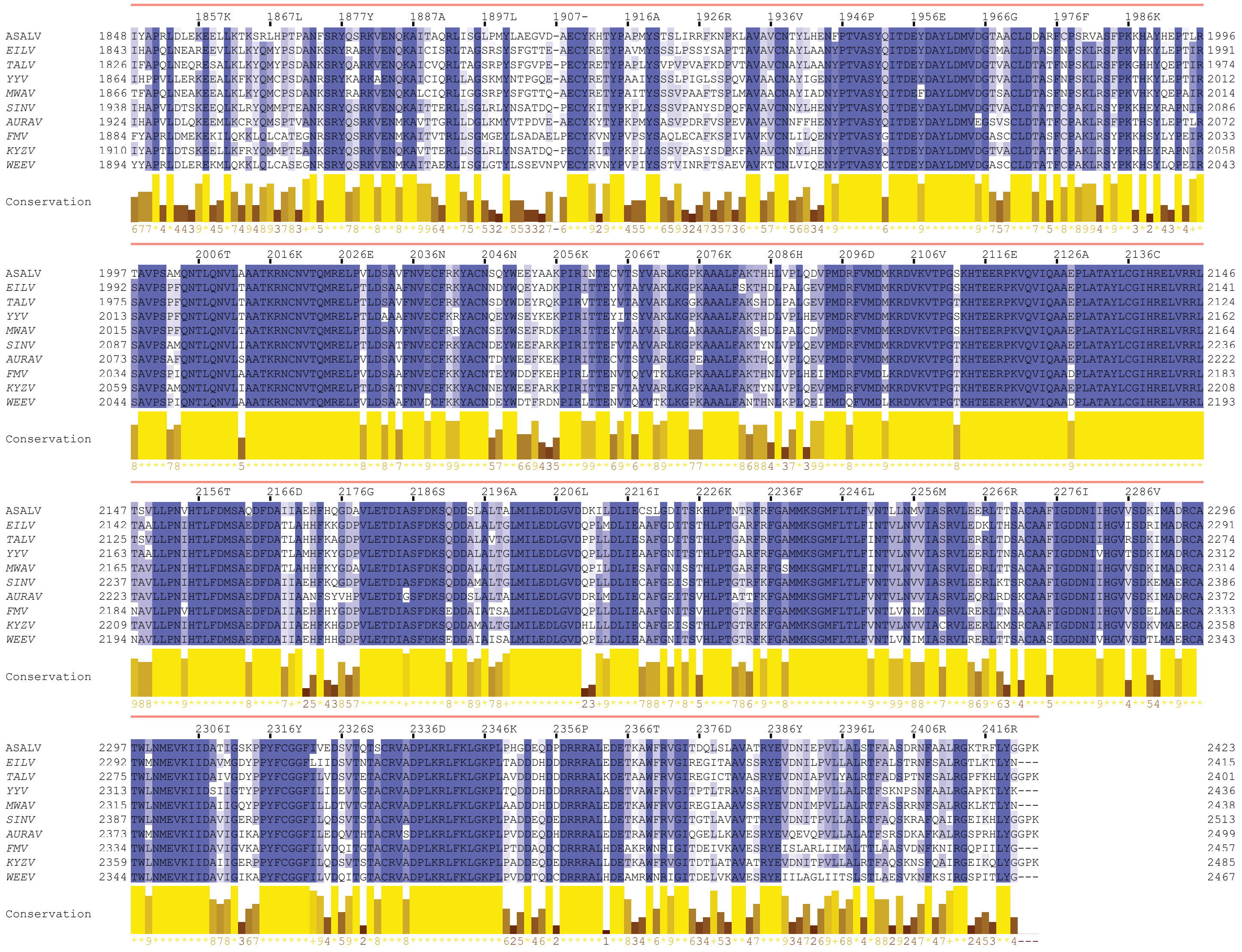

Figure S6 (page 3/3)

SUPPLEMENTAL REFERENCES

1 Wojdyla, J. A., Manolaridis, I., Snijder, E. J., Gorbalenya, A. E., Coutard, B., Piotrowski, Y., Hilgenfeld, R., Tucker, P. A. 2009 Structure of the X (ADRP) domain of nsp3 from feline coronavirus. *Acta Crystallogr D Biol Crystallogr*. **65**, 1292-1300. (10.1107/S0907444909040074)

2 Xu, Y., Cong, L., Chen, C., Wei, L., Zhao, Q., Xu, X., Ma, Y., Bartlam, M., Rao, Z. 2009 Crystal structures of two coronavirus ADP-ribose-1''-monophosphatases and their complexes with ADP-Ribose: a systematic structural analysis of the viral ADRP domain. *J Virol*. **83**, 1083-1092. (10.1128/JVI.01862-08)

3 PDB ID: 6MEA. Hammond, R. G., Schormann, N., McPherson, R. L., Leung, A. K. L., Deivanayagam, C., J., M.A. Characterization of a Novel Macrodomain in the Tylonycteris pachypus Bat Coronavirus HKU4. 10.2210/pdb6mea/pdb

4 Lei, J., Hilgenfeld, R. 2016 Structural and mutational analysis of the interaction between the Middle-East respiratory syndrome coronavirus (MERS-CoV) papain-like protease and human ubiquitin. *Virol Sin*. **31**, 288-299. (10.1007/s12250-016-3742-4)

5 Cho, C. C., Lin, M. H., Chuang, C. Y., Hsu, C. H. 2016 Macro Domain from Middle East Respiratory Syndrome Coronavirus (MERS-CoV) Is an Efficient ADP-ribose Binding Module: CRYSTAL STRUCTURE AND BIOCHEMICAL STUDIES. *J Biol Chem*. **291**, 4894-4902. (10.1074/jbc.M115.700542)

6 Saikatendu, K. S., Joseph, J. S., Subramanian, V., Clayton, T., Griffith, M., Moy, K., Velasquez, J., Neuman, B. W., Buchmeier, M. J., Stevens, R. C.*, et al.* 2005 Structural basis of severe acute respiratory syndrome coronavirus ADP-ribose-1''-phosphate dephosphorylation by a conserved domain of nsP3. *Structure*. **13**, 1665-1675. (10.1016/j.str.2005.07.022)

7 Egloff, M. P., Malet, H., Putics, A., Heinonen, M., Dutartre, H., Frangeul, A., Gruez, A., Campanacci, V., Cambillau, C., Ziebuhr, J.*, et al.* 2006 Structural and functional basis for ADP-ribose and poly(ADP-ribose) binding by viral macro domains. *J Virol*. **80**, 8493-8502. (10.1128/JVI.00713-06)

8 Frick, D. N., Virdi, R. S., Vuksanovic, N., Dahal, N., Silvaggi, N. R. 2020 Molecular Basis for ADP-Ribose Binding to the Mac1 Domain of SARS-CoV-2 nsp3. *Biochemistry*. **59**, 2608-2615. (10.1021/acs.biochem.0c00309)

9 Michalska, K., Kim, Y., Jedrzejczak, R., Maltseva, N. I., Stols, L., Endres, M., Joachimiak, A. 2020 Crystal structures of SARS-CoV-2 ADP-ribose phosphatase: from the apo form to ligand complexes. *IUCrJ*. **7**, 814-824. (10.1107/S2052252520009653)

10 PDB ID: 6YWL. Schroeder, M., Ni, X., Olieric, V., Sharpe, E. M., Wojdyla, J. A., Wang, M., Knapp, S., Chaikuad, A., (SGC), S. G. C. Crystal structure of SARS-CoV-2 (Covid-19) NSP3 macrodomain in complex with ADP-ribose. 10.2210/pdb6ywl/pdb

11 PDB ID: 6WOJ. Kashipathy, M. M., Gao, F. P., Battaile, K. P., Lovell, S., Fehr, A. R. Structure of the SARS-CoV-2 macrodomain (NSP3) in complex with ADP-ribose. 10.2210/pdb6woj/pdb

12 Livingstone, C. D., Barton, G. J. 1993 Protein sequence alignments: a strategy for the hierarchical analysis of residue conservation. *Comput Appl Biosci*. **9**, 745-756. (10.1093/bioinformatics/9.6.745)

13 Nei, M., Kumar, S. 2000 *Molecular evolution and phylogenetics*. Oxford ; New York: Oxford University Press.

14 Jurrus, E., Engel, D., Star, K., Monson, K., Brandi, J., Felberg, L. E., Brookes, D. H., Wilson, L., Chen, J., Liles, K.*, et al.* 2018 Improvements to the APBS biomolecular solvation software suite. *Protein Sci*. **27**, 112-128. (10.1002/pro.3280)
